# Megakaryocyte membrane‐wrapped nanoparticles for targeted cargo delivery to hematopoietic stem and progenitor cells

**DOI:** 10.1002/btm2.10456

**Published:** 2022-11-29

**Authors:** Samik Das, Jenna C. Harris, Erica J. Winter, Chen‐Yuan Kao, Emily S. Day, Eleftherios Terry Papoutsakis

**Affiliations:** ^1^ Department of Chemical and Biomolecular Engineering University of Delaware Newark Delaware USA; ^2^ Delaware Biotechnology Institute University of Delaware Newark Delaware USA; ^3^ Department of Materials Science and Engineering University of Delaware Newark Delaware USA; ^4^ Department of Biological Sciences University of Delaware Newark Delaware USA; ^5^ Department of Biomedical Engineering University of Delaware Newark Delaware USA; ^6^ Helen F. Graham Cancer Center and Research Institute Newark Delaware USA

**Keywords:** biomimetic, endocytosis, gene regulation, hematopoietic stem cells, membrane‐wrapped, targeted delivery

## Abstract

Hematopoietic stem and progenitor cells (HSPCs) are desirable targets for gene therapy but are notoriously difficult to target and transfect. Existing viral vector‐based delivery methods are not effective in HSPCs due to their cytotoxicity, limited HSPC uptake and lack of target specificity (tropism). Poly(lactic‐*co*‐glycolic acid) (PLGA) nanoparticles (NPs) are attractive, nontoxic carriers that can encapsulate various cargo and enable its controlled release. To engineer PLGA NP tropism for HSPCs, megakaryocyte (Mk) membranes, which possess HSPC‐targeting moieties, were extracted and wrapped around PLGA NPs, producing MkNPs. In vitro, fluorophore‐labeled MkNPs are internalized by HSPCs within 24 h and were selectively taken up by HSPCs versus other physiologically related cell types. Using membranes from megakaryoblastic CHRF‐288 cells containing the same HSPC‐targeting moieties as Mks, CHRF‐wrapped NPs (CHNPs) loaded with small interfering RNA facilitated efficient RNA interference upon delivery to HSPCs in vitro. HSPC targeting was conserved in vivo, as poly(ethylene glycol)–PLGA NPs wrapped in CHRF membranes specifically targeted and were taken up by murine bone marrow HSPCs following intravenous administration. These findings suggest that MkNPs and CHNPs are effective and promising vehicles for targeted cargo delivery to HSPCs.

**Abbreviations**
CHMP(s): CHRF microparticle(s)
CHMV(s): CHRF membrane vesicle(s)
CHNP(s): CHRF‐wrapped nanoparticle(s)
CHPPNP(s): CHRF‐wrapped PEG–PLGA nanoparticle(s)
DiD: 1,1‐dioctadecyl‐3,3,3,3‐tetramethylindodicarbocyanine
DLS: dynamic light scattering
HSPC(s): hematopoietic stem and progenitor cell(s)
HUVEC(s): human umbilical vein endothelial cell(s)
IVIS: in vivo imaging system
MFI: mean fluorescence intensity
Mk(s): megakaryocyte(s)
MkMP(s): megakaryocytic microparticle(s)
MkMV(s): megakaryocytic membrane vesicle(s)
MkNP(s): megakaryocyte‐wrapped nanoparticle(s)
MP(s): microparticle(s)
MSC(s): mesenchymal stem cell(s)
MV(s): membrane vesicle(s)
MWNP(s): membrane‐wrapped nanoparticle(s)
NTA: nanoparticle tracking analysis
PEG: poly(ethylene glycol)
PLGA: poly(lactic‐*co*‐glycolic acid)
PMA: phorbol 12‐myrsitate 13‐acetate
NP(s): (PLGA or PEG–PLGA) nanoparticle(s)
siCD34: CD34‐targeting siRNA
siCHNP(s): CHRF‐wrapped, siRNA‐loaded nanoparticle(s)
siNeg: nontargeting siRNA
siNP(s): siRNA‐loaded nanoparticle(s)
TEM: transmission electron microscopy

## 1 INTRODUCTION

Hematopoietic stem and progenitor cells (HSPCs) are predominantly localized in the bone marrow (BM) and possess the ability to self‐renew or differentiate into cells of all blood lineages.^1^ Their ability to differentiate into all blood‐related cells make HSPCs ideal candidates for gene regulation, but enabling effective delivery of nucleic acid cargo to HSPCs is a long‐standing problem whose solution has “formidable promise… that may transform medical practice.”^2^ Thus, by facilitating HSPC‐specific delivery of nucleic acid cargo and other therapeutics, a host of genetic hematological diseases, such as sickle cell anemia and thrombocytopenia, can be ameliorated as diseased HSPCs can be repaired and ultimately differentiate to various lineages of healthy blood cells.^3^, ^4^, ^5^, ^6^

Naked nucleic acids (plasmids, small interfering RNAs [siRNAs], microRNAs [miRNAs]) cannot effectively enter into cells without a carrier or be used clinically because they are rapidly degraded by serum nucleases in vivo, thus resulting in a short blood circulation half‐life.^7^ Current delivery methods using viral vectors (e.g., based on lentivirus or adeno‐associated virus) are hampered by limited loading capacity, poor DNA insertion, and considerable cytotoxicity.^8^ Further, these materials are not well suited for use in HSPCs because the cells lack expression of appropriate adenoviral receptors.^2^ To successfully deliver nucleic acid cargo to HSPCs, a delivery system must specifically target HSPCs while also protecting the cargo. Here, we demonstrate that poly(lactic‐*co*‐glycolic acid) (PLGA) nanoparticles (NPs) can be loaded with nucleic acids and “cloaked” or “wrapped” with megakaryocytic (Mk) membranes that facilitate HSPC‐specific cargo delivery. Membrane‐wrapped NPs (MWNPs) were pioneered by Hu et al. and Parodi et al., who wrapped red blood cell and leukocyte membranes around NPs for improved circulation time and immune evasion, and the concept was expanded upon by Fang et al. who showed that cancer cell MWNPs could facilitate homotypic tumor targeting.^9^, ^10^, ^11^ Here, we exploit this concept to enable targeted cargo delivery to HSPCs.

**FIGURE 1:**
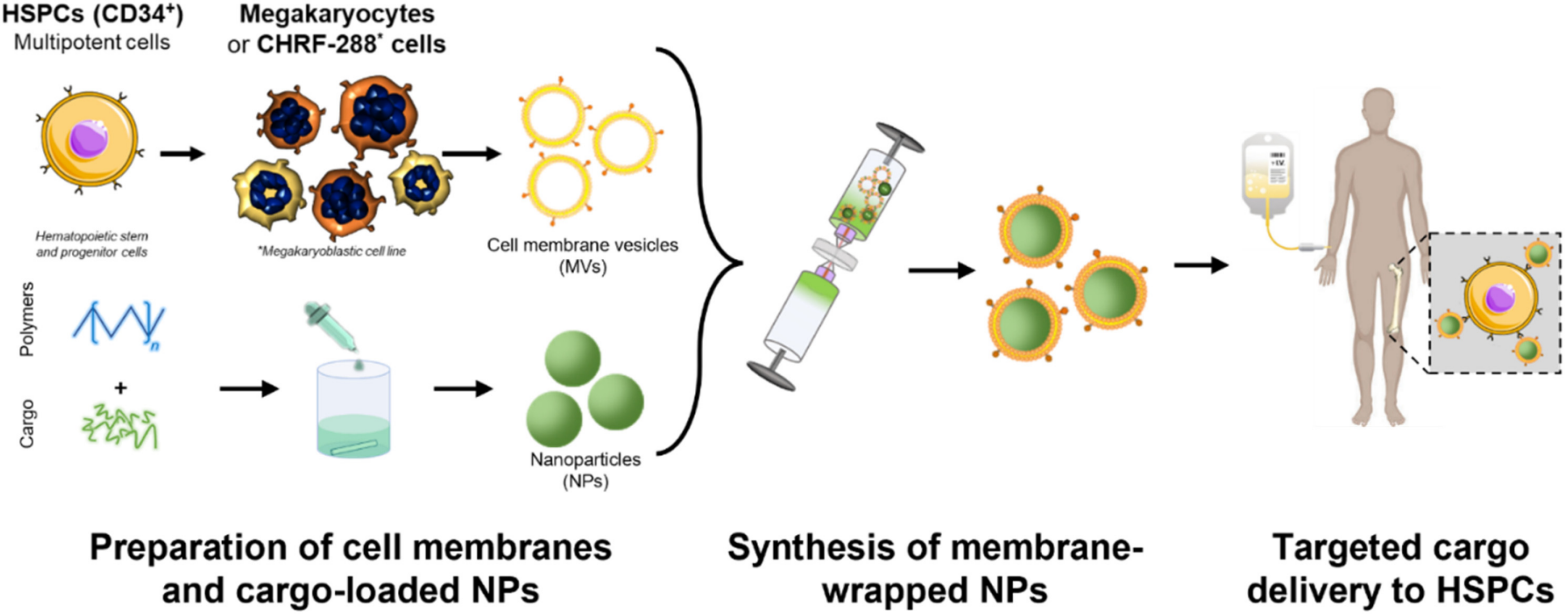
Overview of the synthesis and application of membrane‐wrapped nanoparticles (MWNPs). Megakaryocytic (Mk) membrane vesicles, derived from Mk‐like CHRF‐288 cells, can be wrapped around polymeric nanoparticles (NPs) loaded with desired cargo to produce membrane‐wrapped NPs (MWNPs) that can selectively target, bind, and enter hematopoietic stem and progenitor cells (HSPCs) to deliver their cargo.

Human Mk microparticles (MkMPs) are 100–1000 nm naturally occurring extracellular vesicles^12^ that bud off the cytoplasmic membrane of Mk cells (derived from cultured HSPCs).^13^ They have been shown to specifically target and deliver their cargo to HSPCs, both in vitro^14^, ^15^ and in vivo,^16^ through receptor‐meditated endocytosis and cytoplasmic‐membrane fusion.^14^ Microparticles (MPs) from CHRF cells (termed CMPs) also effectively target and deliver cargo to HSPCs with similar if not better effectiveness.^15^ CHRF cells are a well‐characterized model human megakaryoblastic cell line used to study megakaryopoiesis.^17^ Thus, CHRF membranes are similar to normal Mk membranes, expressing similar membrane glycoproteins. Given the natural targeting ability of MkMPs and CMPs for HSPCs, we hypothesized that membranes extracted from Mk and CHRF cells could be used to produce MWNPs and produce a semisynthetic carrier that can specifically target and deliver cargo to HSPCs (Figure 1). Here, we generated Mk cell‐derived membrane vesicles (MkMVs) and successfully wrapped these vesicles around PLGA NPs loaded with synthetic cargo to form MkNPs. Wrapping was confirmed utilizing transmission electron microscopy (TEM) and nanoparticle tracking analysis (NTA). After incubating fluorophore‐loaded MkNPs with HSPCs, internalization was observed within 24 h primarily via dynamin‐dependent endocytosis, determined using endocytic pathway inhibitors. Further, we demonstrate that these MkNPs preferentially interact with HSPCs over other physiologically‐related cell types of the BM microenvironment,^1^ namely mesenchymal stem cells (MSCs) and endothelial cells (human umbilical vein endothelial cells [HUVECs]). Next, PLGA NPs loaded with siRNA targeting the HSPC marker CD34^18^ were generated using double‐emulsion synthesis and wrapped with MVs from CHRF cells to generate CHRF‐wrapped NPs (CHNPs) loaded with siCD34. Upon delivery to HSPCs, these CHNPs decreased CD34 protein expression levels on HSPCs, indicating successful delivery of functional siRNA cargo.

Finally, we show that CHNPs retain their ability to target HSPCs in vivo. After intravenous administration to Balb/c mice, CHPPNPs (CHRF‐wrapped poly(ethylene glycol) [PEG]‐coated PLGA NPs) exhibited significant, tissue weight‐normalized, localization in the HSPC‐rich BM. Additionally, CHPPNPs were directly taken up by murine HSPCs within the marrow, further confirming the target specificity of CHPPNPs. Control PEG–PLGA NPs exhibited significantly less accumulation in the BM and uptake by HSPCs. Taken together, the data presented here demonstrate that MkNPs and CHNPs are promising vehicles for specific and effective cargo delivery to HSPCs.

## 2 RESULTS

### 2.1 Synthesis and characterization of bare PLGA NPs, MkMVs, and MkNPs

**FIGURE 2:**
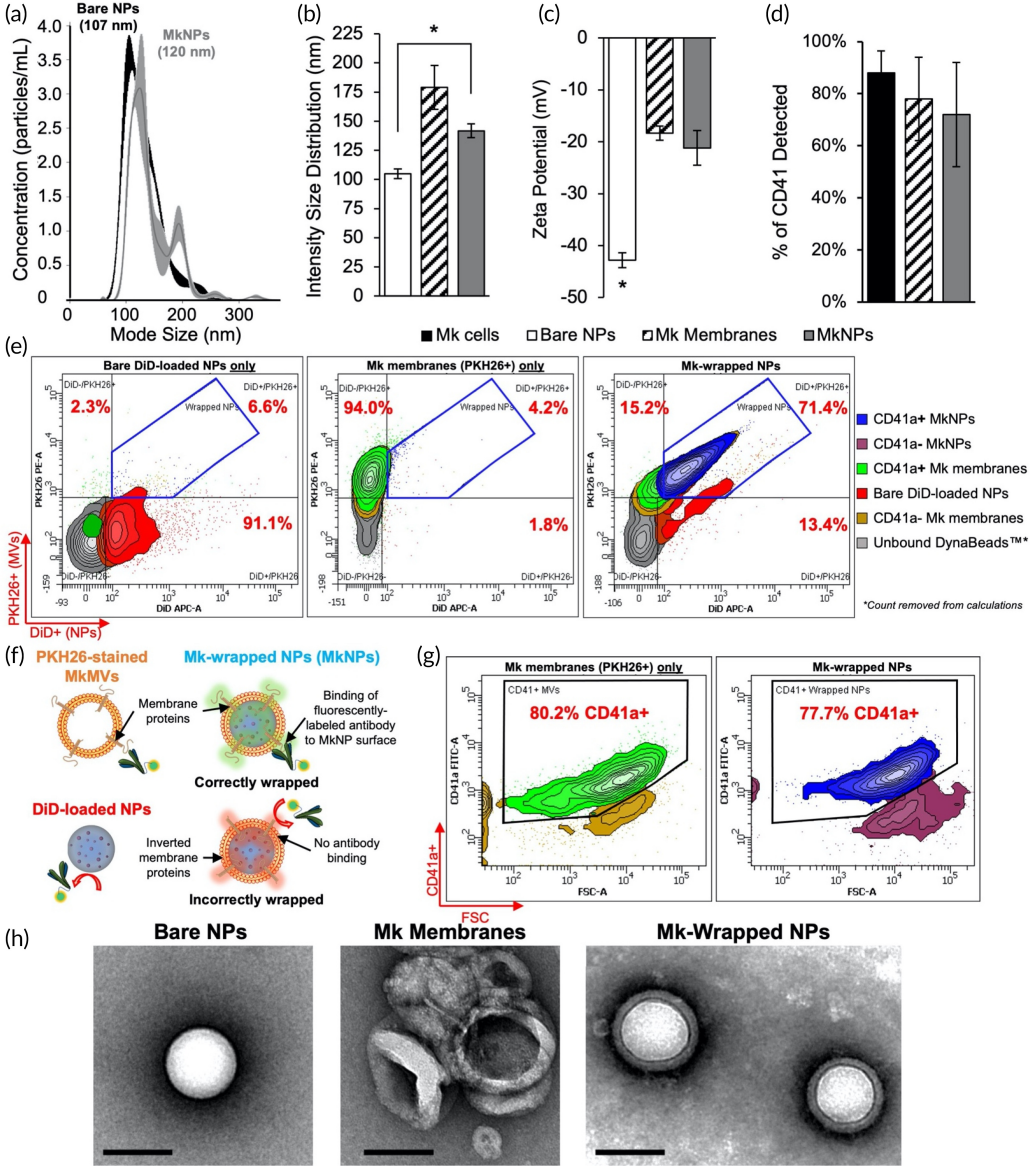
Characterization of megakaryocyte‐wrapped nanoparticles (MkNPs). (a) Representative nanoparticle tracking analysis (NTA) plot showing the shift in size distribution peak upon membrane wrapping. (b) Intensity distribution of the sizes of bare NPs, Mk membranes (MkMVs), and MkNPs measured by dynamic light scattering (DLS). *n* = 3. (c) Zeta potential of bare NPs, MkMVs, and MkNPs. Bare NPs, *n* = 10; Mk membranes and MkNPs, *n* = 8. (d) Percent of CD41a detected on whole cells, MkMVs, and MkNPs by flow cytometry. (e) Flow cytometry was used to gate DiD+, PKH26+ MV‐wrapped NPs (MkNPs) following screening of bare DiD‐loaded PLGA NP‐only (DiD+) and MV‐only samples (PKH26+). (f) Schema of determining proper MV wrapping of NPs via fluorescently conjugated antibodies. (g) CD41a staining of gated MkNPs and MV‐only samples shows high surface expression of CD41a, which indicated proper wrapping and orientation of the membrane proteins on the exterior of the MkNPs. (h) Transmission electron micrographs of bare NPs, MkMVs, and MkNPs. Scale bars = 100 nm. Error bars in (b–d) represent standard error of the mean. *p < 0.05 calculated by one‐way ANOVA with post hoc Tukey

To confirm successful synthesis of MkNPs, several techniques were used to characterize the MkNPs and their precursor components. Both NTA and dynamic light scattering (DLS) reported a mean diameter of bare NPs around 105 nm with an increased size upon wrapping (Figure 2a,b). The NTA readings (Figure 2a) show a 10–15 nm shift in peak size between the bare NPs and the MkNPs and there remains a secondary, smaller peak in the MkNP population that is likely excess membranes in solution. Unlike NTA, DLS cannot fully distinguish excess membranes from MkNPs, resulting in a more dramatic shift between bare NPs and MkNPs (Figure 2b). To further corroborate the size increase is due to wrapping of the particles with membranes, zeta potential measurements revealed that bare PLGA NPs have a zeta potential of about −45 mV, while MkNPs and MkMVs have similar zeta potentials of about −20 mV (Figure 2c). This demonstrates that MkNPs take on the charge of the MVs following successful wrapping. To confirm wrapping efficiency via flow cytometry, PLGA NPs were loaded with DiD fluorophores and the Mk membranes were labeled with PKH26, a strongly lipophilic membrane dye, prior to extrusion. Flow cytometry analysis further determined that greater than 71% of the wrapped sample contained both PKH26 and DiD fluorescence, indicating successful combination of the fluorescently labeled PLGA NPs with the MkMVs following extrusion (Figure 2e).

To determine the proper orientation of the Mk membrane, flow cytometry was used to determine the expression of CD41a (a characteristic Mk membrane marker) detected on whole Mk cells, empty MkMVs, and wrapped MkNPs (Figure 2d,f,g). As CD41a is present on the outer cell membrane of mature Mk cells, successful binding of the fluorescently conjugated antibody to the exposed CD41a membrane protein will indicate proper orientation of the membrane on the PLGA NP surface (Figure 2f).^14^ CD41a levels were similar, with only a slight, but not statistically significant, reduction in CD41a detected on MkNPs and MkMVs compared to whole cells (Figure 2d,g). This confirms that the MkNPs retain the characteristic Mk membrane markers and their proper orientation during the wrapping process. To visualize the membrane layer, bare PLGA NPs, MkMVs, and MkNPs were imaged with TEM (Figure 2h). In these images, bare NPs appear as light‐colored spheres, MkMVs appear as hollow, empty shells, and MkNPs appear as spherical light cores surrounded by thin dark shells, indicative of membrane coating. Through TEM verification of the membrane shells surrounding the entire particle (Figure 2h) and the size and charge changes between bare and wrapped NPs, the NPs were considered successfully wrapped.

### 2.2 MkNPs are readily taken up by HSPCs

**FIGURE 3:**
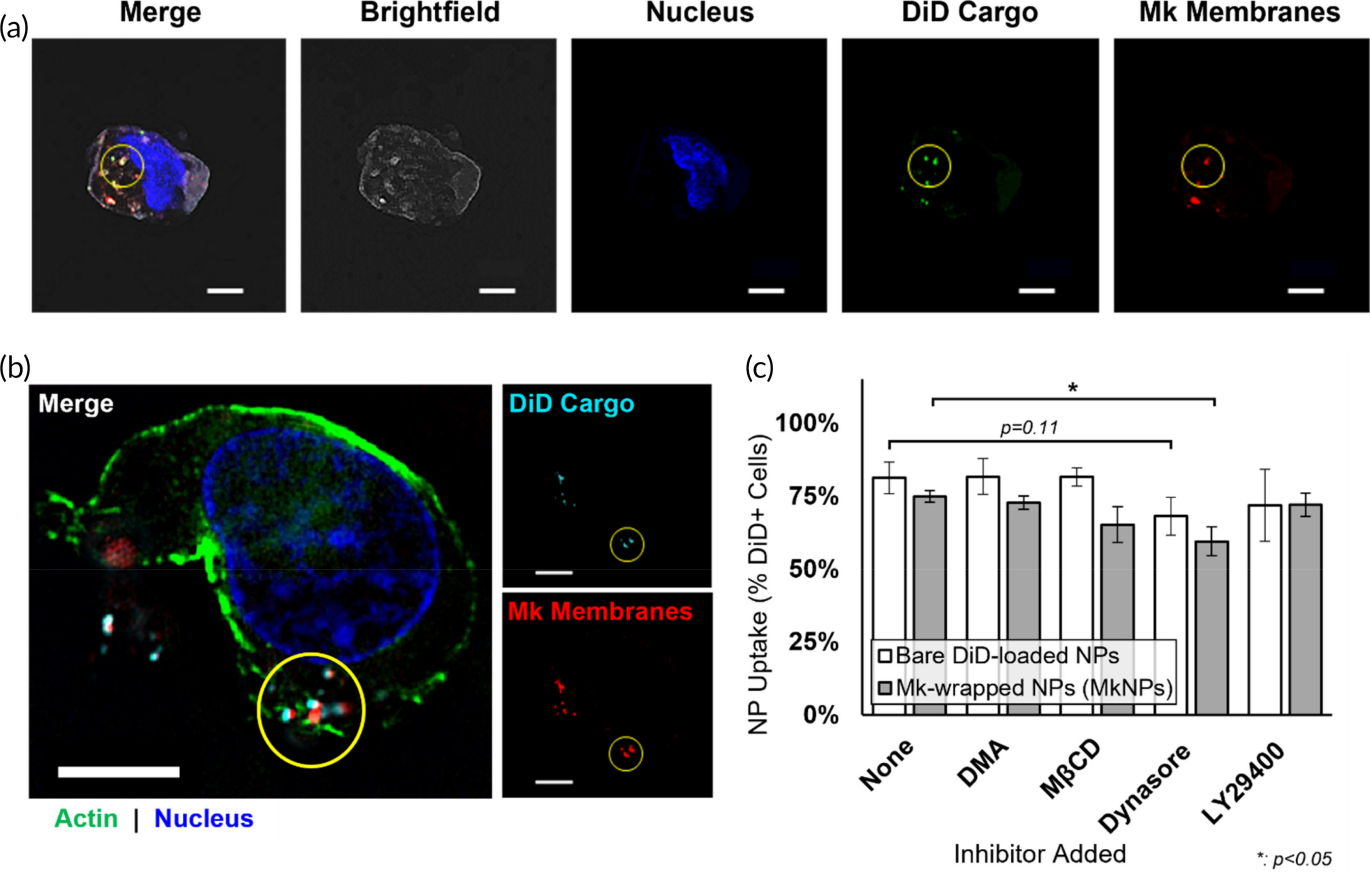
Megakaryocyte‐wrapped nanoparticles (MkNPs) are internalized by hematopoietic stem and progenitor cells (HSPCs) predominantly by dynamin‐dependent endocytosis. (a) Confocal microscopy (Carl Zeiss LSM880) images of an HSPC interacting with MkNPs following 24 h of incubation. Mk membranes are visible by PKH26 staining (red) and NPs were loaded with DiD fluorophores (green). The HSPC nucleus is stained with DAPI (blue). Both PKH26 and DiD signals are present in the HSPC, and colocalization of signals (yellow) indicates the wrapped MkNPs are intact following uptake by HSPCs. Scale bars: 10 μm. (B) HSPCs cultured with MkNPs were fixed and observed by super‐resolution microscopy using Zeiss Elyra PS 1 to visualize internalized MkNPs. The actin cytoskeleton of HSPCs were stained with phalloidin (green) and nuclei were stained with DAPI (blue). MkNPs were labeled with PKH26 (red, membrane marker) and loaded with DiD fluorophores (cyan). Scale bars: 5 μm. For (a) and (b), stills taken from intermediate Z‐stack image are presented and yellow circles indicate internalized MkNPs. (c) Before exposure to either bare DiD‐loaded NPs or MkNPs, HSPCs were preincubated with inhibitors against specific endocytic pathways, including dimethylamiloride (DMA), methyl‐β‐cyclodextrin (MβCD), Dynasore, and LY29400, which block macropinocytosis, lipid raft‐mediated uptake, dynamin‐dependent endocytosis, and macropinocytosis through PI3K, respectively. Uptake of bare NPs and MkNPs was analyzed by assessing the fraction of DiD^+^ cells via flow cytometry following 30 min of incubation, and NP uptake for each inhibitor‐treated culture is shown relative to either bare NP or MkNP uptake in untreated (none) HSPC cultures. Data represent the average of four (bare NPs) and four (MkNPs) biological replicates ± standard error of the mean. *p < 0.05 versus untreated control (Student's *t* test)

We next investigated the interaction between MkNPs and HSPCs in vitro. MkNPs were added to HSPCs that had been cultured for 3 days as described in Section 5.5. For these studies, DiD‐loaded PLGA NPs and the PKH26‐labeled Mk membranes were prepared prior to extrusion. MkNPs are visible by the signal colocalization of the DiD cargo in the NPs and the stained Mk membranes (Figure 3a). Using confocal microscopy, we observed that more than 90% of the HSPCs contained overlapping NP and membrane signals across multiple levels of Z‐stacks after 24 h of incubation, suggesting that MkNPs were internalized intact by the HSPCs. To further confirm that the MkNPs were inside the cells, HSPCs were stained with AF488‐conjugated phalloidin, labeling actin in the outer HSPC membrane and analyzed using super‐resolution structured illumination microscopy (SR‐SIM). As before, minimal MkNP signals were observed along the HSPC membrane at the peripheral Z‐stacks; the colocalization signals were well inside the cells in the medial Z‐stacks, thus confirming that the MkNPs were indeed internalized by HSPCs (Figure 3b). Our analysis further revealed several regions in the HSPC cytoplasm where the NP cargo and membrane signal were colocalized, suggesting the MkNPs were intact following uptake by HSPCs (Figure 3b). Quantitative uptake of MkNPs by HSPCs is presented in Section 2.3.

We also investigated the endocytic pathway by which bare PLGA NPs and MkNPs are taken up by the HSPCs. We followed the experimental design and associated experience from our study using MkMPs.^14^, ^15^ HSPCs were preincubated with endocytic inhibitors including dimethylamiloride (DMA), methyl‐β‐cyclodextrin (MβCD), Dynasore, or LY29400, which block macropinocytosis, lipid raft‐mediated uptake, dynamin‐dependent endocytosis, and macropinocytosis through PI3K, respectively. HSPCs were treated with each of the inhibitors for 45 min and subsequently incubated with either bare NPs or MkNPs as described in Section 5.6. Following incubation with MkNPs, median fluorescence intensity (MFI) of DiD in HSPCs was measured with flow cytometry. Without any inhibitor treatment, roughly 75% of HSPCs exhibited DiD fluorescence after 30 min of incubation with MkNPs (Figure 3c). Among all the inhibitors, Dynasore treatment significantly decreased MkNP uptake by 21% in comparison to the untreated control (Figure 3c), with less than 60% of Dynasore‐treated HSPCs exhibiting DiD fluorescence following incubation with MkNPs. This suggests that uptake of MkNPs involves dynamin‐dependent endocytosis, indicating MkNPs would be taken up primarily through either clathrin‐coated pits or caveosomes. Among HSPCs incubated with bare NPs, Dynasore treatment also reduced NP uptake, but the drop in uptake was less pronounced and not statistically significant compared to their MkNP‐incubated counterpart (Figure 3c).

### 2.3 MkNPs exhibit interaction specificity with CD34 ^+^ HSPCs in comparison to MSCs and HUVECs

To test their target specificity, MkNPs containing DiD were incubated with either CD34^+^ HSPCs or two additional cell types that are physiologically related to HSPCs. MSCs co‐reside in the BM microenvironment with HSPCs, and provide stroma functions for HSPCs.^19^ Mature and immature blood cells, including HSPCs and Mk, enter the systemic circulation through gaps of the endothelium of BM sinusoids^13^, ^20^, ^21^ where they interact with endothelial cells. Here, we used HUVECs as a model endothelial cell. We hypothesized that MkNPs would be preferentially taken up by HSPCs over MSCs and HUVECs. Both flow cytometry and confocal microscopy were used to examine and characterize NP‐cell interactions.

First, we examined the impact of various concentrations of bare PLGA NPs on the viability of each cell type to determine if there was an upper limit in the ratio of NPs to cells that could safely be administered. PLGA NPs are biodegradable and biocompatible, but excess NPs could induce cytotoxicity due to adsorption of cytosolic proteins onto the surface of the NP.^22^ Based on the results of a cytotoxicity assay (Supplemental Figure S1a), we applied a ratio of 40,000:1 NPs per cell as this yielded high NP uptake with minimal tradeoff in viability. As expected, bare DiD NPs were indiscriminately taken up by all examined cells, as >90% of the HSPCs, MSCs, and HUVECs were DiD+ after 8 h of incubation as measured by flow cytometry (Supplemental Figure S1b). This result was confirmed with confocal microscopy (Supplemental Figure S1c), as most HSPC, MSC, and HUVEC cells contained DiD signal, indicative of bare‐NP uptake. DiD signal was found both at the periphery and in the interior of actin‐stained HSPCs, MSCs, and HUVECs, further confirming that bare NPs were taken up indiscriminately by all cells examined (Supplemental Figure S1c).

**FIGURE 4:**
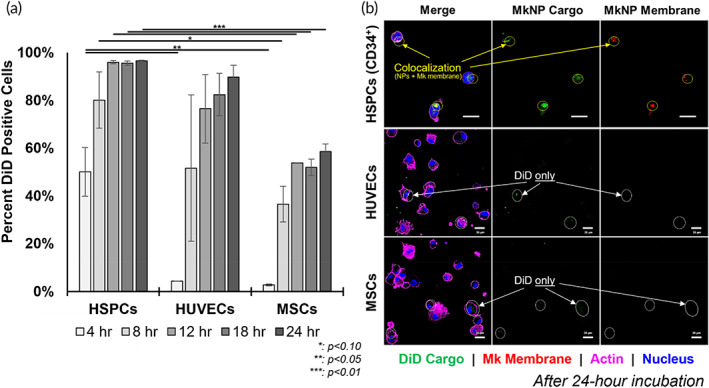
Megakaryocyte‐wrapped nanoparticles (MkNPs) exhibit uptake selectivity by hematopoietic stem and progenitor cells (HSPCs) versus mesenchymal stem cells (MSCs) and human umbilical vein endothelial cells (HUVECs). DiD‐loaded MkNPs were incubated with CD34+ HSPCs, MSCs, or HUVECs in Transwell inserts. (a) Percent DiD positive cells were measured by flow cytometry to reveal MkNP uptake by HSPCs, HUVECs, and MSCs at multiple incubation timepoints (b) Confocal microscopy (Carl Zeiss LSM880) images of HSPCs, HUVECs, and MSCs incubated with DiD‐loaded MkNPs for 24 h. NPs are visualized by their DiD cargo (green) and MkNPs membranes are labeled with PKH26 (red). The cell nuclei are indicated by DAPI stain (blue) and the actin cytoskeleton by phalloidin (purple). MkNPs are found within HSPC cytoplasm but not within nontargeted HUVECs or MSCs. Yellow circles indicate colocalization between DiD and PKH26 signals, which indicate uptake of MkNPs into HSPCs, while white circles indicate intracellular DiD signals without any associated PKH26 signal, indicating possible bare NP or released DiD uptake into HUVECs and MSCs. Scale bars: 10 μm for HSPCs, 20‐μm for HUVECs and MSCs. Error bars are shown as the average ± the standard error of the mean. *p < 0.10, **p < 0.05, ***p < 0.01 (Student's *t* test)

In contrast to bare NPs, MkNPs exhibited differential uptake by HSPCs, MSCs, and HUVECs, supporting our hypothesis that Mk membrane coatings impart target specificity to the NPs. Flow cytometric analysis revealed that, after just 8 h of incubation, over 80% of HSPCs were positive for DiD, indicating MkNP binding or uptake (Figure 4a). At 24 h, most HSPCs displayed colocalization of the DiD (green) cargo with the PKH26 membrane label (red) of the MkNPs (Figure 4b). Colocalization of PKH26 and DiD signals from the MkNPs were found both inside and at the periphery of HSPCs (Figure 4b). In contrast, both flow cytometric and confocal microscopy examination showed that MkNPs were not readily taken up by either HUVECs or MSCs; while some DiD signals were observed within the HUVECs and MSCs, lack of colocalization with PKH26 signal indicates that these DiD signals may be associated with uptake of a small fraction of unwrapped bare NPs or DiD that had been released from the NPs (Figure 4a,b). Based on flow cytometry alone, less than 5% of the MSCs and HUVECs were positive for DiD at the early time points, and less than 50% of the MSC cells were DiD^+^ until approximately 12–18 h following incubation with MkNPs; uptake of MkNPs by HUVEC cells were highly variable until approximately 18 h of incubation (Figure 4a). Thus, we conclude that the MkNPs were not internalized by either HUVECs or MSCs to the same extent as they were internalized by HSPCs, which contained the desired colocalization of DiD and PKH26 signals within the boundaries of the cell. This is consistent with the expectation that recognition of the HSPC target is mediated by surface antigens (notably CD54 [ICAM‐1] and CD18/CD11b [Mac‐1]) expressed on Mk membranes as detailed in the study of Jiang et al. using natively produced MkMPs.^14^ To summarize, we found that coating PLGA NPs with Mk membranes enables their preferential interaction with and uptake by HSPCs versus HUVECs and MSCs.

### 2.4 Mk‐like CHNPs facilitate delivery of siCD34 to HSPCs in vitro

To determine if MWNPs could be viable cargo delivery vehicles, we needed to test if functional cargo could be successfully delivered and deployed to HSPCs. We selected siRNA as a model cargo for our proof‐of‐concept studies. In previous studies, siRNA has proven stable when encapsulated in PLGA NPs.^23^, ^24^, ^25^ We chose to load the NPs with siRNA designed to disrupt the expression of CD34 (siCD34) since CD34 is a characteristic surface marker of immature, undifferentiated HSPCs.^26^ Thus, the effectiveness of siCD34 delivery could be quickly assessed by flow cytometry. Importantly, reducing CD34 expression with siCD34 should have no impact on HSPC viability, as HSPCs display gradually reduced expression of CD34 as they differentiate into different blood cell lineages.^27^, ^28^ For these experiments, nontargeting siRNA (siNeg) was used as a control.

**FIGURE 5:**
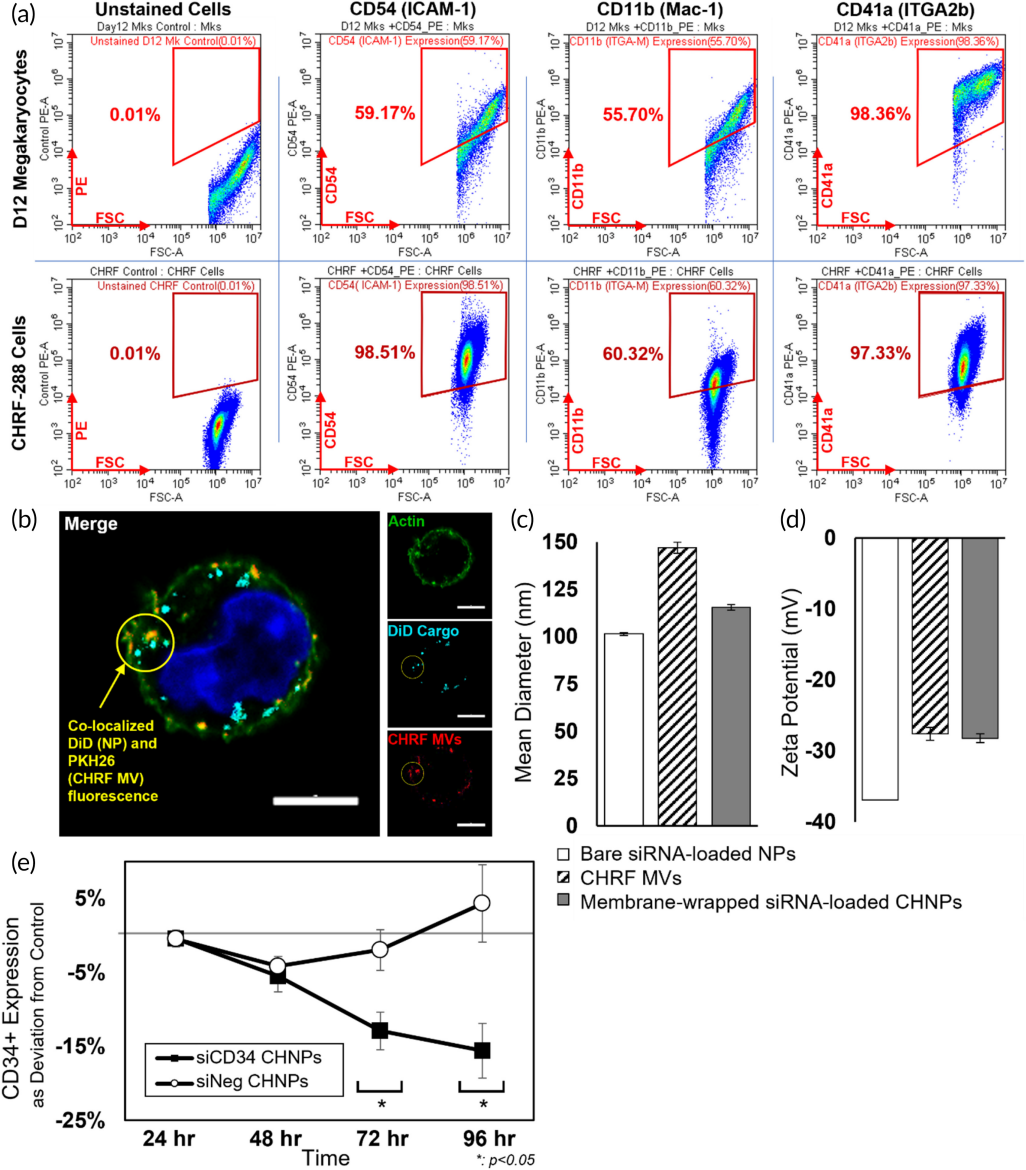
Megakaryocytic membrane vesicles (MkMVs) and Mk‐like CHRF MVs contain surface proteins that facilitate uptake by hematopoietic stem and progenitor cells (HSPCs) and can wrap NPs loaded with functional small interfering RNA (siRNA) cargo for targeted delivery to HSPCs. (a) Flow cytometric analysis showing higher expression of CD54 (ICAM‐1) and CD11b (Mac‐1), which are membrane proteins known to help facilitate uptake in HSPCs, and similar expression of CD41a (ITGA2b), a membrane protein characteristic of mature Mks, in CHRF‐288 (Mk‐like) cells (bottom panel) in comparison to Day 12 cultured Mks (top panel). (b) Confocal microscopy (Carl Zeiss LSM880) images of a Day 3 HSPC interacting with CHRF NPs following 24‐h of incubation. AlexaFluor 488‐stained actin, DiD‐loaded PLGA NPs, PKH26+ CHRF membranes, and nucleus are shown as green, cyan, red, and blue, respectively. Yellow circles indicate colocalized PKH26 and DiD signals within the HSPC, showing successful uptake by HSPCs. Scale bar: 5 μm. (c) Mean diameter of a batch of bare siRNA‐loaded NPs, MVs collected from CHRF cells (CHMVs), and siRNA‐loaded CHRF‐wrapped NPs (CHNPs) measured by nanoparticle tracking analysis showing size increase upon wrapping. (d) Zeta potential of a batch of bare siRNA‐loaded NPs, CHMVs, and siRNA‐loaded CHNPs indicating the surface charge of the particle shifts upon membrane wrapping. (e) CD34+ HSPCs were incubated with CHNPs loaded with siCD34 or nontargeting siRNA (siNeg) for extended periods of time, then CD34 expression was analyzed by flow cytometry. Data are shown as the deviation in CD34 expression from untreated HSPCs. Solid black squares indicate HSPCs treated with siCD34‐loaded CHNPs, and empty circles correspond to (b) siNeg‐loaded CHNPs. Data are shown as the deviation in CD34 expression from untreated HSPCs. Error bars are shown as the average of four replicates ± the standard error of the mean. *p < 0.05 (Student's *t* test)

For these experiments, CHRF‐288‐11 cells, a megakaryoblastic cell line, were used to generate membranes for wrapping siRNA‐loaded NPs as described in Sections 5.4 and 5.7. We first confirmed that the CHRF Mk membranes express the surface proteins (CD54 [ICAM‐1] and CD18/CD11b [Mac‐1])^14^ important for HSPC targeting. As the MVs are generated from the cytoplasmic membranes, CHRF cells and cultured primary Mk cells were immunostained for CD54 and CD11b, as well CD41 expression (to assess the degree of Mk commitment). Interestingly, CHRF cells expressed CD11b and CD54 at higher levels than their Mk counterparts, with 60 and 98% expressing CD11b and CD54, respectively, in comparison to 55 and 59% for Mk cells (Figure 5a). As a visible confirmation of uptake proficiency, DiD‐loaded PLGA NPs were wrapped with PKH26‐stained CHRF membranes and cocultured with HSPCs for 24‐h prior to screening via confocal microscopy. As expected, the CHRF NPs (CHNPs) were effectively taken up by the HSPCs as both DiD and PKH26 signals present with the periphery of the cell (Figure 5b).

Next, the CHNPs were prepared with siCD34 or siNeg. TEM images confirmed successful membrane wrapping around siRNA‐loaded PLGA NPs (Supplemental Figure S2a). Per NTA measurements, siRNA‐loaded CHNPs (siCHNPs) had a mean hydrodynamic diameter of approximately 120 nm (Figure 5c) and a zeta potential of approximately −25 mV (Figure 5d), which matches that of the empty CHMVs (Figure 4d). By comparison, bare siRNA‐loaded NPs had a diameter around 100 nm as determined by TEM and NTA and a zeta potential −35 mV (Figure 5c,d). The difference in the surface charge between DiD‐loaded PLGA NPs (Figure 2c) and siRNA‐loaded PLGA NPs (Figure 5d) is attributed to the 0.1% poly(vinyl alcohol) (PVA) surfactant used during synthesis to help encapsulate siRNA. Notably, this more neutral charge did not hinder the electrostatic interactions that occur between bare NPs and cell membranes to facilitate wrapping.

To quantify the reduction in CD34 expression in HSPCs over time following treatment with siNeg‐CHNPs or siCD34‐CHNPs, flow cytometry data are displayed as percent deviation from the CD34 expression of untreated culture controls. This makes it possible to account for the natural losses in CD34 expression that occur in HSPCs as they mature, and to account for differences in CD34 expression between different HSPC donors.^26^ As expected, HSPCs treated with bare siNeg NPs demonstrated insignificant deviations in CD34 expression from the untreated control (Supplemental Figure S2b). In contrast, siCD34‐loaded bare NPs yielded a significant reduction in CD34 expression, indicating that the siRNA cargo remained functional in HSPCs following delivery (Supplemental Figure S2b). Like the results for bare NPs, we found that HSPCs treated with siNeg‐loaded CHNPs exhibited no loss of CD34 expression, as anticipated, and that HSPCs treated with siCD34‐loaded CHNPs exhibited significant loss of CD34 expression (Figure 5e). Specifically, siCD34‐loaded CHNPs achieved 12.9% CD34 knockdown at 72 h, and 15.6% knockdown at 96 h (Figure 5e). Unwrapped bare NPs achieved 16.9 and 28.9% reductions in CD34 expression at these time points, respectively (Supplemental Figure S2b). The reduced CD34 knockdown of CHNPs relative to bare NPs may be due to suspected cargo loss that occurs during the extrusion technique used to wrap, leading to lower levels of RNA in CHNPs. While the CHNPs yielded slower rates of CD34 disruption in HSPCs than bare NPs, they offer the advantage of specific targeting afforded by the Mk‐like CHRF membranes. This specificity is expected to enable improved delivery of cargo to target cells if implemented in a heterogeneous environment (more than one cell type present) and is explored for in vivo work in Section 2.5.

### 2.5 Intravenously administered CHRF‐membrane wrapped PEG–PLGA NPs in wild‐type Balb/c mice localize to HSPC‐rich tissues and target murine HSPCs in vivo

**FIGURE 6:**
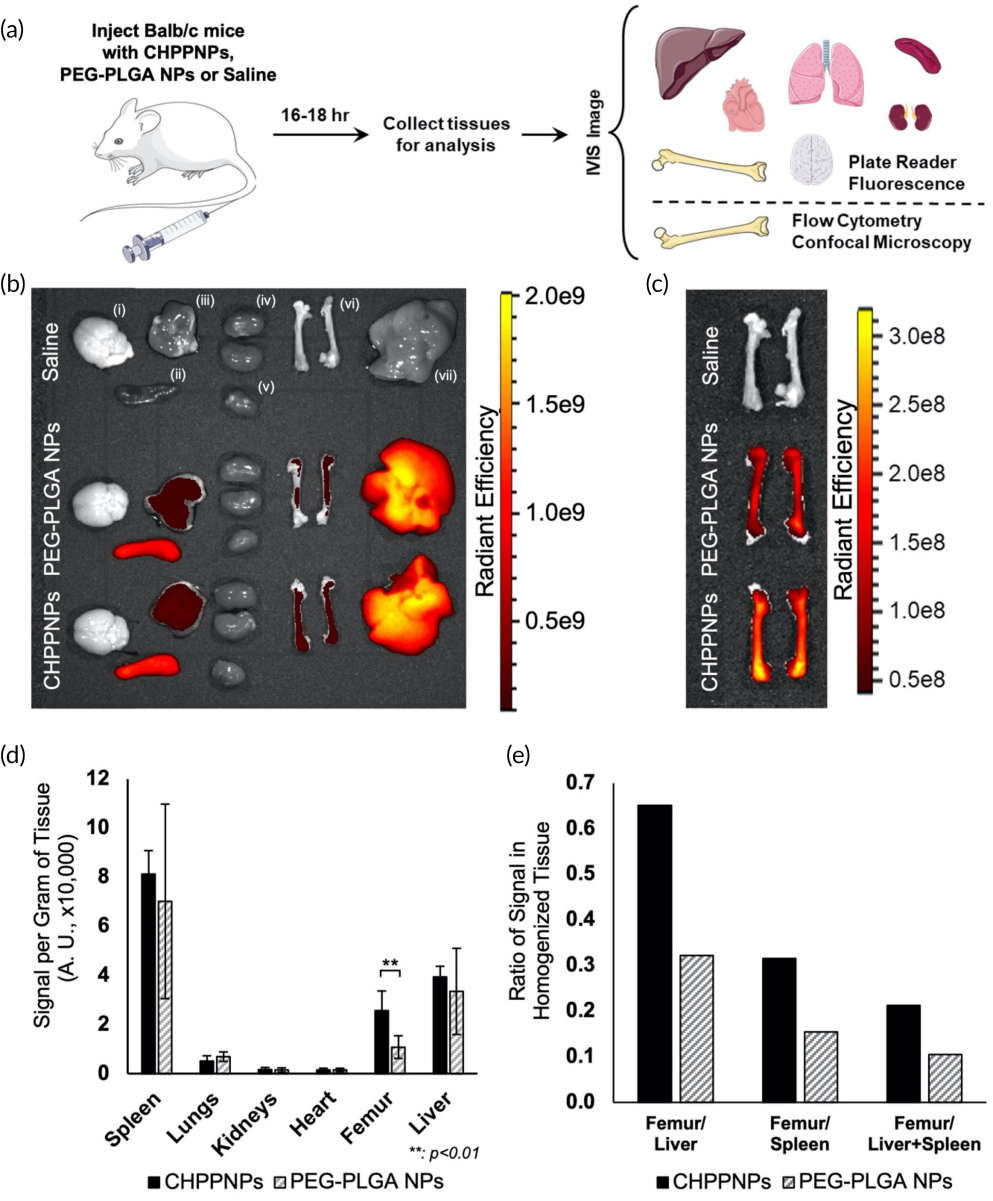
Intravenously administered CHRF‐wrapped PEG–PLGA nanoparticles (CHPPNPs) largely localize to hematopoietic stem and progenitor cell (HSPC)‐rich bone marrow in vivo. (a) Mice were injected with CHPPNPs, poly(ethylene glycol) (PEG)–NPs, or saline and humanely sacrificed after 16–18 h. Tissues (liver, heart, lungs, brain, spleen, kidneys, and femurs) were collected and in vivo imaging system (IVIS) imaged. Bone marrow was collected from one femur and used for flow cytometry and confocal microscopy analysis while all other tissues were homogenized in PBS and the signal measured by fluorescent plate reader analysis. (b) Representative IVIS image of various tissues: (i) brain, (ii) spleen, (iii) lungs, (iv) kidneys, (v) heart, (vi) femurs, and (vii) liver, of mice treated with saline, DiD‐loaded PEG–PLGA NPs, and DiD‐loaded CHPPNPs. (c) Representative IVIS image of femurs from each group comparing DiD signal between groups. (d) Averaged fluorescence signal per gram of homogenized tissue as measured by plate reader. (e) Ratio of signal in one femur to liver, spleen, or the combination of the two. **p < 0.01, calculated by one‐way ANOVA. PEG NPs: *n* = 6; wrapped NPs: *n* = 5. Errors bars show standard error of the mean.

After demonstrating robust interaction of CHNPs with HSPCs in vitro, we wanted to test if HSPC targeting remains valid in vivo using 10‐ to 12‐week‐old female Balb/c mice. For these studies, the core of the particle was altered to be comprised a 1:3 ratio of PLGA‐*b*‐PEG:PLGA instead of the solely PLGA core used in the prior experiments. PEG is widely used for stealth coating of circulating particles. Saline, DiD‐loaded PEG–PLGA NPs (112 nm average diameter by NTA measurement, −30 mV zeta potential), or their CHRF‐membrane‐wrapped counterparts (referred to as CHPPNPs, 126 nm average diameter as calculated by NTA, −30 mV zeta potential) were administered intravenously. After 16–18 h, femurs, lungs, heart, liver, spleen, kidneys, and brain were excised via necropsy (Figure 6a). Tissues were fluorescently imaged using an in vivo imaging system (IVIS) to examine DiD presence (Figure 6b) and the fluorescence signal in the femurs of mice treated with CHPPNPs was greater than that of PEG–PLGA NPs (Figure 6C). However, as IVIS is a more qualitative technique that views from the top down, the bone or tissue components may be blocking some of the signal and it is important to quantify the results by another method. Therefore, each tissue was weighed, homogenized, and DiD signal quantified by fluorescence plate reader analysis (Figure 6d). The average signal in the flushed BM from a femur of CHPPNP‐treated mice was 2.4 times higher than that in the BM of PEG–PLGA‐treated mice, indicating the potential of the membrane‐wrapped system to enhance cargo delivery to the BM. When further comparing the two NP treatments, we found that the ratio of BM signal in one femur to that in the liver, spleen, or the combination of the two was over twice as high for CHPPNPs than PEG–PLGA (Figure 6e). This indicates that CHPPNPs outperform PEG–PLGA NPs in terms of their ability to localize to BM versus the major clearance organs. Importantly, we did not observe any change in the tissue morphology of CHPPNP‐treated mice (Supplemental Figure S3), indicating the CHPPNPs are biocompatible under the conditions, administered dose, and 18‐h timepoint examined.

**FIGURE 7:**
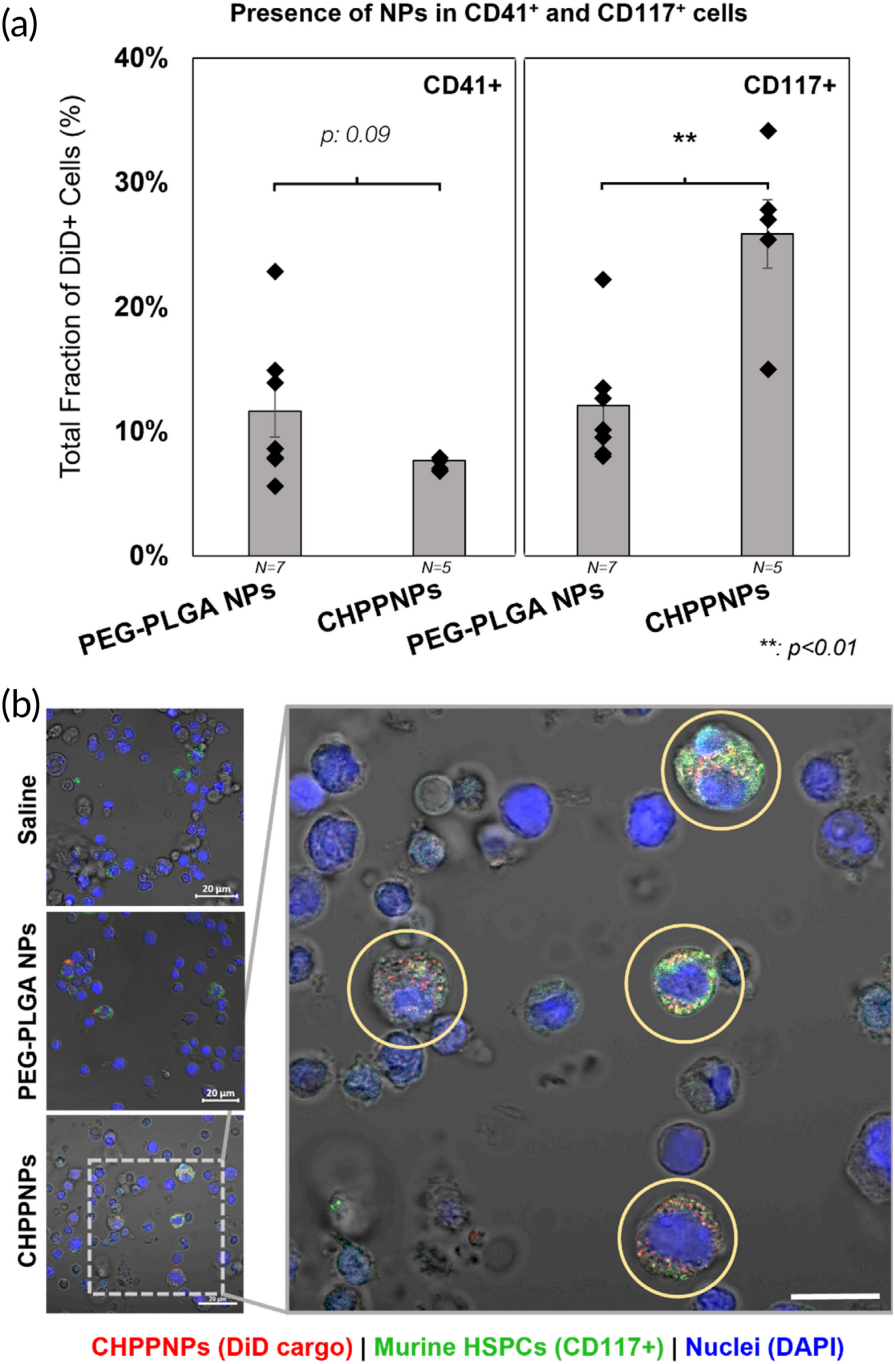
DiD presence in CD41a+ and CD117+ bone marrow cells of mice treated with poly(ethylene glycol)–poly(lactic‐*co*‐glycolic acid) (PEG–PLGA) nanoparticles (NPs) and CHPPNPs. (a) Bone marrow cells were flushed from the femurs of mice treated with a saline control (not shown), DiD‐loaded PEG–PLGA NPs, or DiD‐loaded CHPPNPs and stained for CD41a (platelet‐associated cells) and CD117 (murine hematopoietic stem and progenitor cells [HSPCs]). Flow cytometry indicates a significant difference between PEG–PLGA NPs and CHNPs in the fraction of CD117+ cells that contain DiD signal, while a nonsignificant decrease in the fraction of DiD‐containing CD41a+ cells was seen, possibly demonstrating a level of HSPC affinity afforded by the membrane in contrast to other cell types. Each black diamond corresponds to one mouse. (b) Flushed bone marrow cells were stained for CD117 (green) and DAPI (cell nuclei; blue) and imaged by confocal microscopy to visualize the presence of NPs, indicated by DiD (red). Scale bars: 20 and 10 μm in magnified image. Error bars are shown as the average of seven (bare NPs) or five (CHPPNPs) biological replicates ± the standard error of the mean. **p < 0.01 (Student's *t* test)

As the BM is comparatively HSPC‐rich, we flushed and collected the BM cells from the femur for further analysis via flow cytometry and confocal microscopy. Whole BM cells were stained with fluorescently conjugated anti‐CD117 and anti‐CD41a antibodies, which correspond to murine HSPCs and Mk‐associated cells, respectively. CD117 was specifically chosen to determine if HSPC targeting by MkNPs and CHNPs observed in vitro translated in vivo, while CD41a would allow us to assess if the MkNPs/CHNPs could target their murine Mk counterparts. After staining, the whole BM cells were gated for the presence of CD117 and CD41 against unstained controls, and the subpopulations of CD117^+^ and CD41^+^ cells were further screened and gated for the presence of DiD against the saline‐treated mouse control. From our flow cytometry and confocal microscopy analysis, a significantly larger fraction of CD117^+^ marrow cells from mice treated with the CHPPNPs contained DiD fluorescence than their unwrapped PEG–PLGA NP counterparts (Figure 7a, Supplemental Figure S4,b); nearly 26% of CD117^+^ marrow cells from CHPPNP‐treated mice were DiD‐fluorescent in comparison to under 13% of bare NP‐treated mice. The higher fraction of DiD^+^ CD117^+^ marrow cells from CHPPNP‐treated mice could indicate that the Mk membrane affords some HSPC targeting ability to the NP, in agreement with our in vitro findings. Surprisingly, there was no significant difference in the fraction of DiD^+^ CD41 marrow cells, which implies that the Mk‐derived CHRF membrane does not provide any targeting to Mks. This further supports the suitability of using MkNPs or CHPPNPs for targeted delivery to HSPCs.

To confirm these results, the whole BM cells were fixed onto coverslips and stained for CD117 for analysis via confocal microscopy to look for DiD fluorescence and colocalization of DiD and CD117 signals, which would indicate NP‐containing murine HSPCs (Figure 7b, Supplemental Figure S4a,b). As shown in Figure 7b, marrow cells from CHPPNP‐treated mice contained various degrees of DiD fluorescence, which is consistent with the findings of Figure 6. In addition, there was a greater degree of colocalization of the DiD signal with CD117‐stained cells, indicating that the CHRF membranes enable HSPC‐target specificity. Impressively, CHPPNPs were almost exclusively bound to CD117^+^ cells within the flushed BM, with virtually no presence of CHPPNPs observed on CD117^−^ cells (which are not HSPCs). This further supports that the contention that CHPPNPs maintain their specificity and preferentially interact with HSPCs in vivo. PEG–PLGA NPs exhibited less overall accumulation in the marrow and less specificity for CD117^+^ cells than the CHPPNPs. Coupling these phenomena, we conclude that CHPPNPs synthesized using CHRF membranes are a promising candidate for targeted cargo delivery to HSPCs in vivo.

## 3 DISCUSSION

PLGA has emerged as an attractive choice for harboring therapeutics for drug delivery, largely due to its stability and broad biocompatibility.^29^, ^30^, ^31^, ^32^ PLGA NPs may be loaded with a broad range of therapeutic molecules, from small molecules to miRNAs and DNA with minimal loss of functionality.^24^, ^25^, ^33^, ^34^ However, bare or PEG‐coated PLGA NPs administered in vivo lack tissue tropism, thus limiting the potency of the encapsulated therapeutic.^32^, ^35^ PLGA and other NPs can be modified to target specific tissues by coating them with different types of cell‐derived membranes.^36^ For example, Xu et al. used HepG2 liver cancer cell membranes to target doxorubicin‐loaded PLGA NPs to liver cancer; Kroll et al. wrapped B16‐F10 murine melanoma membranes around adjuvant‐loaded PLGA cores to activate an anticancer immune response by presenting the membrane‐bound antigens to dendritic cells, and; Jiang et al. created hybrid NPs wrapped with a mixture of erythrocyte and MCF‐7 breast cancer cell membranes to both target tumors and extend circulation time.^37^, ^38^, ^39^ Beyond PLGA carriers, many other materials have been used to create MWNPs.^36^, ^40^ This includes synthetic and naturally occurring polymers that offer stability, biocompatibility, and easy manipulation, as well as metallic NP cores that offer unique imaging and photo‐responsive properties.^41^ In this work, PLGA was chosen due to its wide application in nanomedicine, ability to encapsulate both hydrophilic and hydrophobic cargo, ease of synthesis, and proven use in membrane‐wrapped carrier systems. Here, we demonstrate that wrapping PLGA NPs in Mk membranes can enable targeted cargo delivery to HSPCs.

We have previously demonstrated that MkMPs can recognize and fuse with HSPCs for HSPC‐specific cargo delivery.^14^, ^15^ As MkMPs bud off the cytoplasmic cell membrane, Mk membranes and vesicles preserve the membrane proteins, including those that mediate cell‐specific binding. We thus expected that PLGA or PEG–PLGA NPs wrapped in Mk membranes would maintain the HSPC recognition and target specificity of their MkMP counterparts. Accordingly, MkNPs could combine the benefits of HSPC targeting specificity with the versatility of PLGA NPs as a nanocarrier for therapeutic delivery. The in vitro studies reported here confirm that MkNPs can facilitate cargo delivery to HSPCs while minimizing interactions with other cell types present in the BM environment. This specificity and tropism is maintained in vivo, as HSPC‐rich tissues and HSPCs isolated from these tissues contained a significant presence of CHPPNPs several hours following their intravenous administration. One potential confounder is that our studies are based on fluorescence of DiD, which could be released from the NPs while they are in circulation. While the microscopy images (in vitro: Figures 3a,b, 4, and 5, in vivo: Figure 7b) do show colocalization of the cargo and membranes in targeted HSPCs, this does not rule out the possibility that released dye is also in circulation. To address this potential question, future work could explore using fluorescent PLGA to affirm that the signal is not from released dye.

Receptor‐mediated interactions play an important role in the ability of MkMPs (and thus of Mk membranes) to recognize and target HSPCs with great specificity.^14^ Here, we show that MkNPs enter HSPCs largely via dynamin‐dependent endocytosis. Next, we chose MSCs and HUVECs to probe the targeting ability and HSPC specificity of MkNPs as was previously done with MkMPs.^14^ Unwrapped NPs were readily taken up by all cell types, but MkNPs were effectively and specifically internalized by HSPCs with very few HUVECs and MSCs displaying signs of MkNP uptake. By extension, MkNPs also exhibit targeting of HSPCs in vivo, further supporting the potential for MkNPs to be efficacious HSPC‐specific cargo delivery vehicles. In addition to demonstrating MkNPs are specific for HSPCs, our studies also showed that MkNPs can support siRNA delivery to HSPCs. This opens the door for other nucleic acids or therapeutic agents to be delivered to HSPCs in the future via an MWNP system.

While we demonstrated robust and preferential uptake of MkNPs into HSPCs both in vitro and in vivo, additional work will be needed to further validate MkNPs as a full‐fledged targeted drug delivery system. Our data indicate that siRNA‐loaded CHPPNPs successfully disrupted expression of CD34 upon delivery to HSPCs. However, as we were unable to quantify the specific amount of siRNA encapsulated within the CHNPs, our dosing strategy was based on CHPPNP particle counts and particle to HSPC ratios rather than the total amount of siRNA delivered to each cell. Future studies will need to quantitate the amount of siRNA contained within each NP, the release kinetics of the siRNA from the NPs, and the total amount of siRNA delivered to each target cell. Additionally, using siRNA‐loaded CHPPNPs in vivo with a murine disease model could further showcase their potential for facilitating HSPC‐specific RNA‐based therapies for a variety of genetic hematological diseases.^42^, ^43^, ^44^ As mentioned earlier, future studies could also explore the compatibility of MkNPs or CHNPs with various classes of therapeutic cargo rather than the model (non‐therapeutic) cargo we used as a proof of concept here.

## 4 CONCLUSION

In conclusion, we have shown that MkNPs and CHNPs specifically and robustly interact with hard‐to‐transfect HSPCs both in vitro and in vivo and can be used to deliver functional nucleic acid cargo to HSPCs. With further development and optimization, these tools may be used to enable the treatment of a broad spectrum of blood disorders.

## 5 MATERIALS AND METHODS

### 5.1 Materials and antibodies

All chemicals were purchased from Fisher Scientific and Sigma Aldrich unless otherwise indicated. HSPC and Mk culture media, BIT 9500, was purchased from STEMCELL Technologies. All recombinant human interleukins (rhIL‐3, rhIL‐6, rhIL‐9, rhIL‐11), stem cell factor (rhSCF), and thrombopoietin (rhTPO) were from PeproTech. For the CHRF media, heat inactivated fetal bovine serum (FBS) was from Millipore Sigma. Frozen G‐SCF mobilized human peripheral blood CD34^+^ cells were obtained from the Fred Hutchinson Cancer Center (Seattle, WA) from anonymized healthy donors. MSCs and culture media were from Lonza Tech. HUVEC (endothelial) cells and culture media were from Millipore Sigma. The Avanti Mini Extruder and associated consumables (filter supports, polycarbonate membranes) were from Avanti Polar Lipids. Fluorescently conjugated antibodies (monoclonal mouse IgG2b allophycocyanin (APC)‐tagged antihuman CD34) were from BD Lifesciences. All siRNAs (siCD34 and nontargeting siNeg) were purchased from Dharmacon.

### 5.2 Mk and CHRF‐288‐11 cultures

Human CD34^+^ D0 (Day 0) cells were cultured in IMDM supplemented with 20% BIT 9500, rhSCF, rhTPO, rhIL‐3, rhIL‐6, rhIL‐11, and human LDL to produce Mks and incubated in a hypoxic (5% O_2_, 5% CO_2_) humidified (95% rH) environment at 37°C as described by Panuganti et al.^26^ After a Day‐5 media exchange, Day 7 CD61^+^ cells (Mks) were enriched using anti‐CD61 magnetic microbeads (Miltenyi Biotec) and were cultured in IMDM containing 20% BIT 9500. rhSCF, rhTPO, human LDL, and nicotinamide as described by Panuganti et al.,^26^ and incubated in a 20% O_2_, 5% CO_2_, and 85% rH environment at 37°C. On Day 12, Mks were collected, and membranes were isolated as explained in Section 5.3.

For the siRNA and in vivo experiments, CHRF‐288‐11 cells^17^ were used in lieu of Mk cells due to their comparatively faster culture and lower cost. When treated with phorbol 12‐myrsitate 13‐acetate (PMA),^15^, ^17^ CHRF cells differentiate into Mk cells that also produce microparticles. Cells were initially expanded in CHRF medium (IMDM, 10% FBS, Na_2_CO_3_, and antibiotic/antimycotic). Next, CHRF cells were seeded (200,000/ml) in static T flasks with CHRF media supplemented with 0.1 nmol PMA. After a 3‐day incubation, the adherent and suspension CHRF‐PMA cells were harvested to produce CHRF MVs (CHMVs) following the same collection procedure as Mk cells described in Section 5.3.

To characterize specific membrane protein expression of mature Mks and CHRF cells, 100 μl of cell culture (approximate density: 200‐500 k cells/ml) were incubated with 5 μl (all monoclonal mouse IgG2b anti human) PE anti‐CD11b, PE anti‐CD41a, or PE anti‐CD54 antibody (BD Lifesciences) at 4°C for 15 min, and 200 μl filtered PBS was added to each sample prior to analysis by flow cytometry (Beckman Coulter CytoFLEX S). Expression was gated against unstained Mk or CHRF controls.^13^, ^14^

### 5.3 Preparation and isolation of Mk and CHMVs

Mk and CHRF MVs were generated from mature Day 12 Mk cells or Day 3, PMA treated CHRF cells, prepared as described in Section 5.2. To generate the membranes for NP wrapping, we used an estimate of 2000–4000 MVs generated per cell. Briefly, cells were collected, washed, dyed with PKH26 (PKH26 Red Fluorescent Cell Linker Kit for General Cell Membrane Labeling‐Sigma) and excess dye was quenched with 1% bovine serum albumin (BSA). Next, cells were washed and suspended in a hypotonic lysis buffer (20 mM Tris HCl, 10 mM KCl, 2 mM MgCl_2_) containing 0.5% v/v protease inhibitor cocktail (p8340, Sigma) prior to disruption using two differently sized Dounce homogenizers with tight‐fitting pestles (Kimble) as described.^11^ The entire solution was subjected to 30 passes with each pestle before spinning down at 3200 × *g* for 5 min at 4°C. The supernatants were collected and spun down at 20,000 × *g* for 20 min at 4°C, after which the pellets were discarded or used for protein analysis, and subsequently centrifuged at 100,000*g* for 90 min at 4°C.^11^ The supernatant was discarded, and pelleted cell membranes were resuspended in Biology Grade Molecular Water (Corning). To generate MkMVs, isolated membranes were extruded through a 400 nm polycarbonate membrane (Avanti) for 11 passes (Avanti Mini Extruder). Final MkMV and CHMV concentration was measured using NTA (Malvern NanoSight NS300) for subsequent characterization and preparation of MWNPs.

### 5.4 Synthesis and characterization of PLGA NPs and MkNPs

For initial in vitro studies, PLGA NPs prepared by single emulsion synthesis were loaded with DiD fluorophores as a model cargo to allow visualization of cargo delivery to HSPCs. Briefly, 50:50 PLGA (LACTEL Absorbable Polymers) (inherent viscosity: 0.67 dl/g, molecular weight: 39.5 kDa), was dissolved in acetone at 2 mg/ml and DiD was added at a concentration of 12 μM. This mixture was then added dropwise to water in a 1:3 ratio, and the solvent allowed to evaporate overnight under continuous stirring at 800 rpm at room temperature. The resultant DiD‐loaded PLGA NPs were purified by 10 kDa molecular weight cut‐off (MWCO) centrifugal filtration (Sigma) at 3200*g* for 30 min at room temperature.

Prior to membrane wrapping, the size and concentration of bare NPs and extruded MkMVs were determined by NTA. Each sample was diluted in molecular biology‐grade water to an optimal concentration of 1 × 10^8^ to 5 × 10^9^ particles per milliliter. Three technical replicates for each sample were analyzed and each video was analyzed by the NTA software to reveal the mean and mode diameter and estimate of the sample concentration. Based on the concentration of bare NPs and MkMVs determined by NTA, the NPs and MVs were premixed together at a wrapping ratio of 1:2 NPs:MkMVs and coextruded through a 400 nm polycarbonate membrane (Avanti Polar Lipids) for 7–11 passes within a heating block set at 55°C.^11^ The MkNP suspension was then centrifuged at 20,000*g* for 20 min at 4°C to pellet, and the supernatant was removed. MkNPs were resuspended in water for characterization and further use. The size and zeta potential of bare PLGA NPs, empty MkMVs, and wrapped MkNPs were determined with an Anton Paar LiteSizer500. NTA was utilized to determine the diameter and concentration of MkNPs. To visualize the morphology of bare NPs, MkMVs, and MkNPs, samples were placed on TEM grids, stained with 2% uranyl acetate, dried, and examined by TEM (Carl Zeiss Libra 120). To quantify the presence of CD41a on cells, 100 μl of cell culture was stained with monoclonal FITC mouse IgG2b antihuman CD41a with the protocol described in Section 5.2 and analyzed by flow cytometry (BD FACS Aria II). Before quantifying CD41a presence on MkMVs and MkNPs, 0.1 μl streptavidin‐conjugated T1 Dynabeads (Invitrogen) were incubated with 2 μl biotin‐conjugated monoclonal mouse IgG1 antihuman CD41a (Invitrogen) prior to adding to each sample. The volume of each sample was brought up to 500 μl with filtered PBS, and the samples were mixed via rotation (LabQuake Shaker) for 2 h at room temperature. Next, the samples were washed with filtered PBS containing 0.1% BSA, and the washed DynaBead‐coupled MkMVs and MkNPs were resuspended in 300 μl filtered PBS. Finally, the samples were incubated with 10 μl monoclonal mouse IgG2b FITC antihuman CD41a antibody (BD Lifesciences) at room temperature for 15–30 min prior to analysis via flow cytometry (BD FACS Aria II). Scatter plots using forward and side scatter were used to exclude cell debris and dead cells and create a main live‐cell population gate. Signal was adjusted to reduce overflow between channels and additional gates were created based on unstained cells and single stain controls. MkNPs were selected from a population containing both PKH26 and DiD; presence of CD41a on the MkNPs was determined from this subpopulation. Analysis presented is the average of four replicates.

### 5.5 Incubation of HSPCs with MkNPs for microscopic analysis of cellular interactions

Cells from Day 3 CD34^+^‐cell cultures were resuspended in culture medium (IMDM +20% BIT serum substitute +1% antibiotic–antimycotic (αα), 100 ng/ml rhTPO and rhSCF) and added to Transwell membrane wells (0.4 μm pore size, Costar 3470) in a 24‐well tissue culture plate (Falcon 353047, Corning) as described.^13^, ^14^, ^15^ After 30 min, MkMVs, bare NPs, or MkNPs were added in each Transwell membrane insert, and the plate was centrifuged at 600*g* for 30 min to maximize interaction between the particles and HSPCs. The plate was subsequently incubated at 5% CO_2_, 20% O_2_, 95% relative humidity (rH), and 37°C. After incubating for 24 h, cells were stained with AF488 phalloidin and DAPI to visualize the actin cytoskeleton and nucleus, respectively, and transferred to a μ‐slide eight chambered coverslip with poly‐l‐lysine (Ibidi 80826). Live cells were examined using confocal microscopy with a 40× oil objective (Carl Zeiss LSM880 multiphoton confocal microscope). For SR‐SIM (Carl Zeiss Elyra PS1), cells were fixed with 4% paraformaldehyde (PFA) in PBS, stained with Alexa Fluor 488 (AF488)‐phalloidin, mounted on slides with SlowFade with DAPI (Thermo Fisher) and visualized using a 63× oil objective. The DiD cargo, PKH26‐labeled membrane, phalloidin‐stained HSPC cytoskeleton, and DAPI‐stained HSPC nuclei were imaged in their respective channels. For assessing the interaction between CHRF‐wrapped DiD‐loaded PLGA NPs and HSPCs, the previous protocol was repeated with PKH26‐stained CHRF membranes used in lieu of Mk membranes. To analyze HSPC‐CHRF NP interaction, the culture was fixed with 4% PFA in PBS following 24‐h of incubation, stained with AF488‐phalloidin, mounted onto slides with SlowFade with DAPI, and imaged with a 63× oil objective (Carl Zeiss LSM880).

### 5.6 Use of endocytosis inhibitors to examine the route of MkNP uptake by HSPCs

To elucidate the mechanism by which bare PLGA NPs and MkNPs might enter HSPCs, cells from Day 3 CD34^+^‐cell cultures placed in centrifuge tubes were pretreated with various endocytosis inhibitors prior to incubation with bare NPs or MkNPs according to our previous study.^14^ Specifically, 10 μM DMA (Sigma), 80 μM Dynasore (Sigma), 5 mM MβCD (Sigma), or 50 μM LY29400 (Sigma Aldrich) were incubated with the cells to inhibit macropinocytosis, dynamin‐dependent (clathrin‐dependent) endocytosis, lipid raft‐mediated endocytosis, and macropinocytosis, specifically blocking PI3K, respectively. Cells and inhibitors were vortexed for 30 s and incubated at 37°C, 20% O_2_, 85% humidity for 45 min prior to administering either bare NPs or wrapped MkNPs. The samples were vortexed in the tubes for 30 s, transferred to 12‐well plates and incubated at 37°C for 30 min. After incubation, cells were washed with PBS and pelleted at 500 × *g* for 5 min. Cells were resuspended in 300 μl PBS and analyzed by flow cytometry (BD FACS Aria II). The intensity of the DiD dye within the NPs was detected using the APC channel, and the MFI determined with three measurements for each sample. The MFI of cells that were not exposed to NPs was used to set a gate such that the percent of DiD positive cells in each treatment group could be established. A paired student *t* test was performed to compare the intensity in samples treated with each inhibitor to the intensity in samples not treated with any inhibitor.

### 5.7 Interaction of MkNPs with CD34 ^+^ (HSPCs), MSCs, and endothelial cells, and the cytotoxicity assay

CD34^+^ (i.e., HSPCs) were thawed and prepared as described in Section 5.5. MSCs (Lonza) and HUVECs (Sigma) were cultured in non‐differentiating serum‐supplemented MSC growth media (Lonza) and VEGF‐supplemented endothelial cell growth media (ATCC), respectively, and expanded at 20% O_2_, 5% CO_2_, and an 85% rH environment at 37°C in their respective media and passaged several times prior to incubation with NPs and MkNPs. Both MSCs and HUVECs were seeded at roughly 40–60% confluence and ~130,000 of the prepared Day 0 CD34^+^ cells were seeded in the 0.4‐μm Transwell inserts before adding DiD‐labeled PLGA NPs 2 h later. Five wells corresponding to five timepoints (4‐, 8‐, 12‐, 18‐, and 24‐h) were prepared for each of the HSPC, MSC, and HUVEC cultures, and cells were collected for measurement just once from each well to minimize disruption to the interactions between particles and cells. A ratio of approximately 40,000 particles (determined by NTA) per viable cell for both the bare NPs and MkNPs was used, as determined by final yields of synthesized NPs and MkNPs.

To analyze DiD signal in NP‐treated MSCs and HUVECs, cells were detached from the wells using Accutase (Sigma) and resuspended in 200 μl of the respective cell media. For confocal microscopy, approximately 100 μl of the cell suspension was seeded onto separate poly‐l‐lysine coated coverslips and incubated at 37°C for 15 min; 50 μl was reserved for flow cytometry. Seeded cells were subsequently fixed using 4% PFA and washed several times using filtered PBS, and the fixed (unpermeabilized) cells were incubated with AF488‐conjugated phalloidin for 30 min to stain actin in the cytoskeleton.^45^ After several washes with filtered PBS, the fixed slides were mounted using mounting media (Slow Fade with DAPI) and sealed with coverslips. Slides were imaged using confocal microscopy with a 40× oil objective (Carl Zeiss LSM880) and measured using their respective fluorescent channels.

To assess the optimal dose of bare NPs to cell, unwrapped DiD‐labeled PLGA NPs were incubated with CD34^+^ cells, MSCs, and HUVECs at ratios of 1 × 10^4^, 1×10^5^, and 1 × 10^6^ NPs per cell in a 12‐well plate (2 biological replicates) and stored at 37°C, 20% O_2_, 85% humidity. After 24 h, cells were removed from their respective wells, washed, and incubated in filtered PBS containing 0.1% ethidium homodimer for 15 min at 37°C to assess viability. Cells were then analyzed and gated for live and dead cells using size and fluorescence under the PE channel. DiD NP uptake was tabulated by measuring the number of DiD^+^ live cells under the APC channel.

### 5.8 Generation of siRNA‐loaded CHNPs

To encapsulate hydrophilic siRNA cargo in PLGA NPs, a previously established double emulsion water‐in‐oil‐in‐water procedure was adapted.^46^ Briefly, the first water phase was created by adding 0.4 nmol siCD34 or siNeg to 100 μl of 1% PVA in deionized (DI) water. The siRNA/PVA solution was then added dropwise, while stirring, to 1 ml of a 4 mg/ml PLGA‐in‐acetone solution that was prepared by dissolving 50:50 PLGA (LACTEL Absorbable Polymers) (inherent viscosity: 0.67 dl/g) in acetone. The mixture was stirred for 5 min at 800 rpm to create a water‐in‐oil emulsion that was then added dropwise while stirring to 0.1% aqueous PVA at a 1:3 ratio, to produce the water‐in‐oil‐in‐water solution. The solvent was evaporated overnight under continuous stirring at 800 rpm and the resultant NPs were purified by 50 kDa MWCO centrifugal filtration (Sigma) at 3200*g* for 30 min at 4°C. siRNA‐NPs were wrapped as described in Section 5.4 using CHRF membranes and the final product characterized by TEM, zeta potential, and NTA.

### 5.9 Treatment of HSPCs with siRNA‐loaded NPs for analysis of CD34 knockdown

Frozen CD34^+^ D0 cells were thawed and prepared in a modified culture medium as described in Section 5.5. The modified medium did not include rhTPO, as it induces differentiation of HSPCs into a Mk lineage, which would accelerate CD34 downregulation and would thus confound results. Approximately 130,000 CD34^+^ cells were seeded into multiple 0.4 μm Transwell inserts at 37°C for 2 h prior to incubation with either bare siNPs or wrapped siCHNPs. Approximately 40,000 NPs per cell of both bare siNPs and siCHNPs were dosed into their respective wells after seeding, along with polybrene (Sigma) (1:2000 v/v, polybrene: medium) to enhance NP interaction with cells and aid transport across the cell membrane.^47^ After 24 h, cells were aspirated from the Transwell inserts, spun at 300*g* for 10 min, and resuspended in fresh modified culture media (containing IMDM +20% BIT serum substitute +1% antibiotic–antimycotic (αα), and 100 ng/ml rhSCF only) to remove any excess siNPs and siMkNPs from suspension.

To measure the cells' CD34 expression at each time point (24, 48, 72, and 96 h), 75 μl of the sample and a negative control consisting of solely CD34 cells in the modified culture medium were incubated with 5 μl monoclonal APC mouse IgG2b antihuman CD34 antibody (BD Lifesciences) at 4°C for 15 min. Then, 220 μl of filtered PBS was added and the APC intensity of each sample was measured against the untreated negative control using flow cytometry. Reduction in CD34 expression was calculated by taking the difference in the percentage of CD34^+^ cells between the experimental conditions (siCD34‐ or siNeg‐loaded NPs and CHNPs) and the untreated negative control at each time point. An unpaired student's *t* test was performed to compare both siCHNP conditions to the untreated negative control.

### 5.10 Synthesis of membrane‐wrapped PEG–PLGA NPs for in vivo biodistribution

For in vivo experiments, PEG–PLGA NPs were used as a biocompatible base for the MkNPs. PEG–PLGA methyl ether (Nanosoft Polymers) was dissolved in dichloromethane at 2 mg/ml and mixed at a 1:3 ratio with the acetone‐PLGA solution (Section 5.4). DiD was added at a concentration of 12 μM and this mixture was then added dropwise to 0.1% PVA in a 1:3 ratio, and probe sonicated on ice for 60 s. The solvent was allowed to evaporate overnight under continuous stirring at 800 rpm at room temperature and the resultant PEG–PLGA NPs were purified by 10 kDa MWCO centrifugal filtration (Sigma) at 3200*g* for 30 min at room temperature. NPs were left unwrapped or wrapped with CHRF membranes following the steps of Section 5.4 and characterized by NTA, zeta potential, and TEM. These particles are referred to as CHPPNPs, (CHRF‐wrapped PEG–PLGA NPs) as they are different from the CHNPs described above.

### 5.11 Assessing biodistribution of CHPPNPs to determine targeting of murine HSPCs

Animal studies were performed in accordance with institutional guidelines under a protocol approved by the Institutional Animal Care and Use Committee. Then, 1 mg of CHPPNPs suspended in 100 μl sterile saline was intravenously administered to Balb/c mice via the tail vein; equivalent amounts of unwrapped (bare) PEG–PLGA NPs and saline were used as controls. After 16–18 h, the mice were euthanized via CO_2_ asphyxiation and cervical dislocation, and the following tissues were excised and added to PBS with 1% antibiotic–antimycotic: femurs, lungs, liver, spleen, kidneys, heart, and brain.

Immediately post‐extraction, whole tissues were imaged using a PerkinElmer IVIS Lumina III with excitation and emission filters of 640 nm/710 nm to capture DiD signal in tissues. After imaging, tissues were weighed and homogenized in PBS using Potter‐Elvehjem PTFE pestle and glass tubes (Sigma Aldrich). Fluorescence signal from three wells containing 100 μl each of homogenized tissue (excluding femurs) were measured by Synergy H1 Microplate Reader (Biotek) (ex/em 640 nm/675 nm) and averaged to calculate signal per gram of tissue. To measure signal in BM, the ends of the femurs were trimmed, and the marrow flushed using a 28G needle with 100 μl PBS repeatedly until marrow was visibly removed. Solution was added to a single well and read by microplate reader with the same settings as the tissues. Weight used in signal calculations of the femurs was based on the average change in weight of the end‐trimmed femurs pre‐ and post‐flushing of the marrow. Tissues from representative mice were paraffin embedded, sectioned, and stained with hematoxylin and eosin (H&E). Slides were imaged with a light microscope to examine any morphological changes.

To extract the BM cells for flow cytometry and microscopy, each femur was placed in a clean petri dish and scraped clean of any other tissue (muscle, connective tissue, etc.) and rinsed with 1× PBS containing antibiotic–antimycotic. Next, the epiphyses were trimmed, and a 3‐ml syringe equipped with a 20G needle and filled with the RPMI‐FBS tissue storage buffer was used to flush the BM out of the bone. The BM cells were subsequently added atop a 30‐μm pre‐separation filter (Miltenyi Biotec). Finally, additional RPMI tissue storage buffer was added to the petri dish, and the evacuated femur was crushed. The buffer containing the crushed femur was transferred to the pre‐separation filter, and the marrow cells were spun at 300*g* for 10 min and washed with PBS.

The extracted and washed BM cells were subsequently analyzed via flow cytometry and confocal microscopy. For the flow cytometry measurements, 50 μl of the marrow cell suspension was incubated with 2.5 μl of fluorescently conjugated monoclonal rat IgG2b 2B8 anti‐mouse CD41a and anti‐mouse CD117 antibodies (Becton Dickinson) and incubated at 4°C for 15 min prior to analysis; separate aliquots of each sample were used with each antibody. For the preparation of slides for confocal microscopy, 100 μl of each BM cell suspension was seeded on glass coverslips pretreated with poly‐l‐lysine and subsequently fixed with 4% PFA (Electron Microscopy Services). Next, the seeded and fixed coverslips were blocked with blocking buffer containing 10% normal donkey serum (Abcam) and 3% BSA for 1 h at room temperature. After blocking, each coverslip was rinsed with filtered PBS, and 200 μl blocking buffer containing 5 μl of fluorescently conjugated monoclonal rat IgG2b 2B8 anti‐mouse CD117 (BioLegend) was added to each coverslip; the coverslips were stained for 2 h in the dark at room temperature. The stained coverslips were rinsed with filtered PBS, mounted onto slides containing SlowFade mounting media with DAPI (Invitrogen), sealed with nail polish, and stored at 4°C prior to imaging. Each sample was imaged using the Carl Zeiss LSM880 confocal microscope and analyzed using the Zeiss Zen software.

## AUTHOR CONTRIBUTIONS

**Samik Das:** Investigation (equal); methodology (equal); visualization (equal); writing – original draft (equal); writing – review and editing (equal). **Jenna Harris:** Investigation (equal); methodology (equal); visualization (equal); writing – original draft (equal); writing – review and editing (equal). **Erica Winter:** Investigation (equal); methodology (equal); writing – original draft (equal). **Chen‐Yuan Kao:** Methodology (equal). **Emily Day:** Conceptualization (equal); funding acquisition (equal); investigation (equal); methodology (equal); supervision (equal); visualization (equal); writing – review and editing (equal). **Eleftherios Terry Papoutsakis:** Conceptualization (equal); funding acquisition (equal); investigation (equal); methodology (equal); supervision (equal); visualization (equal); writing – review and editing (equal).

## CONFLICT OF INTEREST

The authors have a patent pending related to the MkNP technology under international PCT application number PCT/US2019/063685.

### PEER REVIEW

The peer review history for this article is available at https://publons.com/publon/10.1002/btm2.10456.

## Supporting information


**APPENDIX S1** Supporting Information

## Data Availability

The data that support the findings of this study are available from the corresponding author upon reasonable request.
