# Supplementary material for: Megakaryocyte membrane‐wrapped nanoparticles for targeted cargo delivery to hematopoietic stem and progenitor cells
Source: Bioeng Transl Med. 2022 Nov 29;8(3):e10456. doi: 10.1002/btm2.10456 (PMC10189472; doi:10.1002/btm2.10456)
Supplement: Supplementary file 1 — APPENDIX S1 Supporting Information [file BTM2-8-e10456-s001.docx]

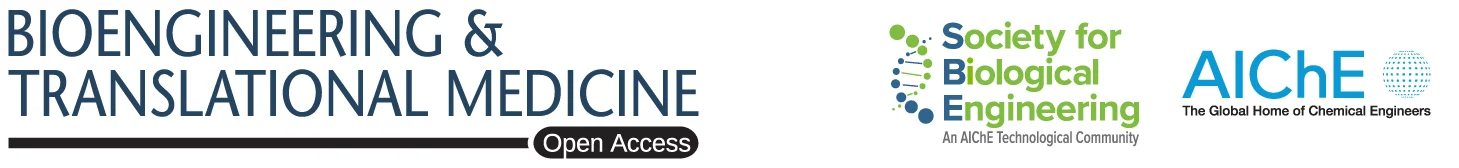


Supplementary Materials for

**Megakaryocyte membrane-wrapped nanoparticles for targeted cargo delivery to hematopoietic stem and progenitor cells**

Samik Das, Jenna C. Harris, Erica J. Winter, Chen-Yuan Kao, Emily S. Day*, E. Terry Papoutsakis**

*Corresponding author. Email: [emilyday@udel.edu](mailto:emilyday@udel.edu)

**Corresponding author. Email: [epaps@udel.edu](mailto:epaps@udel.edu)

**This PDF file includes:**

**Figure S1:** Analysis of the cytocompatibility and target specificity of unwrapped DiD-loaded NPs.

**Figure S2:** TEM microscopy of siRNA-loaded NPs and CD34 expression in HSPCs exposed to siRNA-loaded bare PLGA NPs.

**Figure S3:** Representative hematoxylin and eosin (H&E) staining of tissues indicate no toxicity

**Figure S4:** DiD signal presence in flushed bone marrow cells of mice treated with saline, PEG-PLGA NPs, or CHNPs

Figure S1.


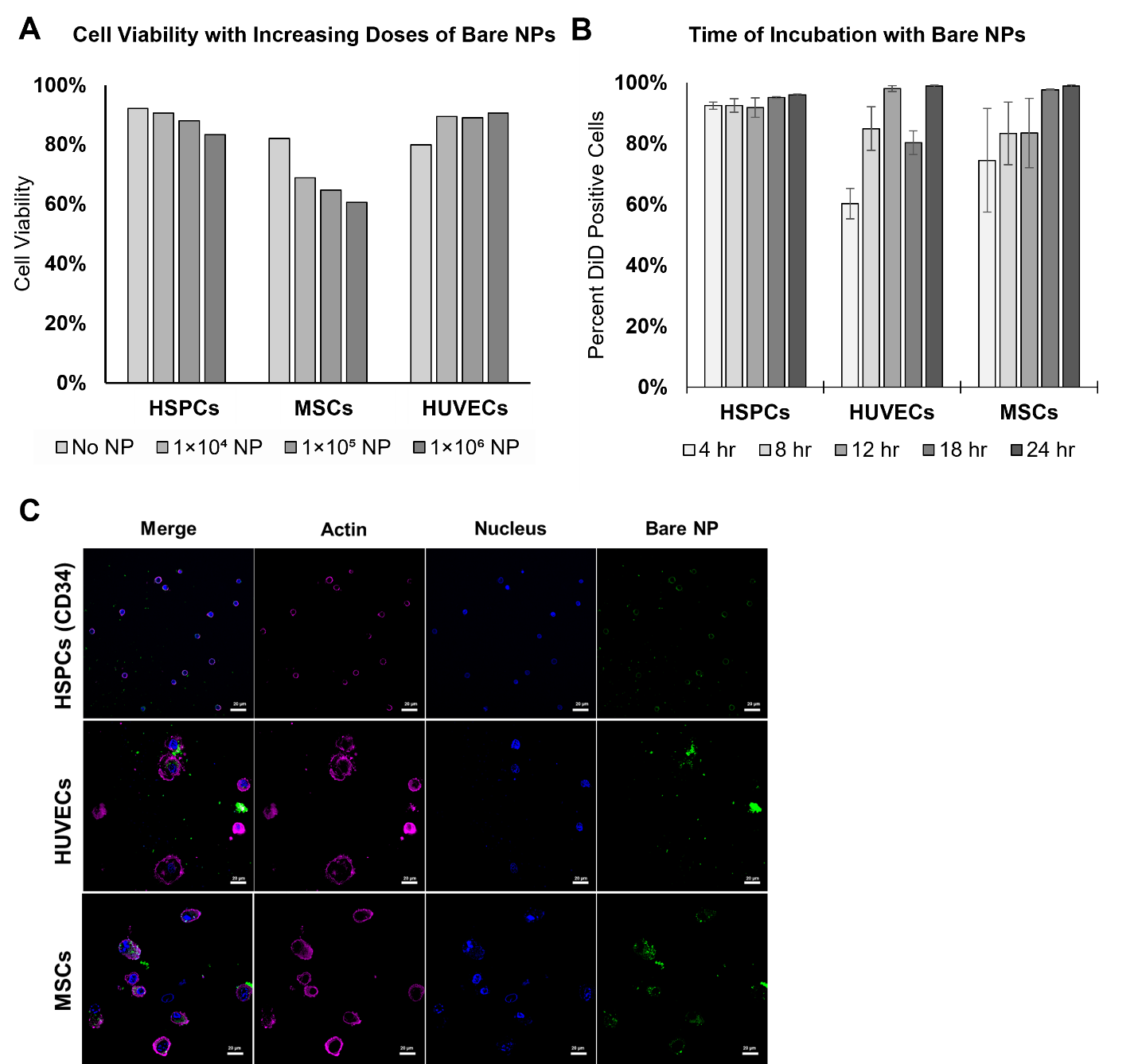


**Supplemental Figure S1. Analysis of the cytocompatibility and target specificity of unwrapped DiD-loaded NPs.** (A) PLGA NPs were added to cells at increasing doses to ensure cell viability was maintained. (B) Flow cytometry examination of bare NP uptake by HSPCs, HUVECs, and MSCs as determined by measurement of DiD signal following NP culture with the cells for different amounts of time. Bare NPs exhibit no preferential uptake by different cell types. (C) Confocal microcopy images (Zeiss LSM880) of bare NPs interacting with HSPCs, HUVECs, or MSCs. Blue indicates cell nuclei (DAPI), green indicates bare NP cargo (DiD), and purple indicates cell actin cytoskeleton (phalloidin). Scale bars: 10-µm for HSPCs, 20-µm for HUVECs and MSCs.

Figure S2.


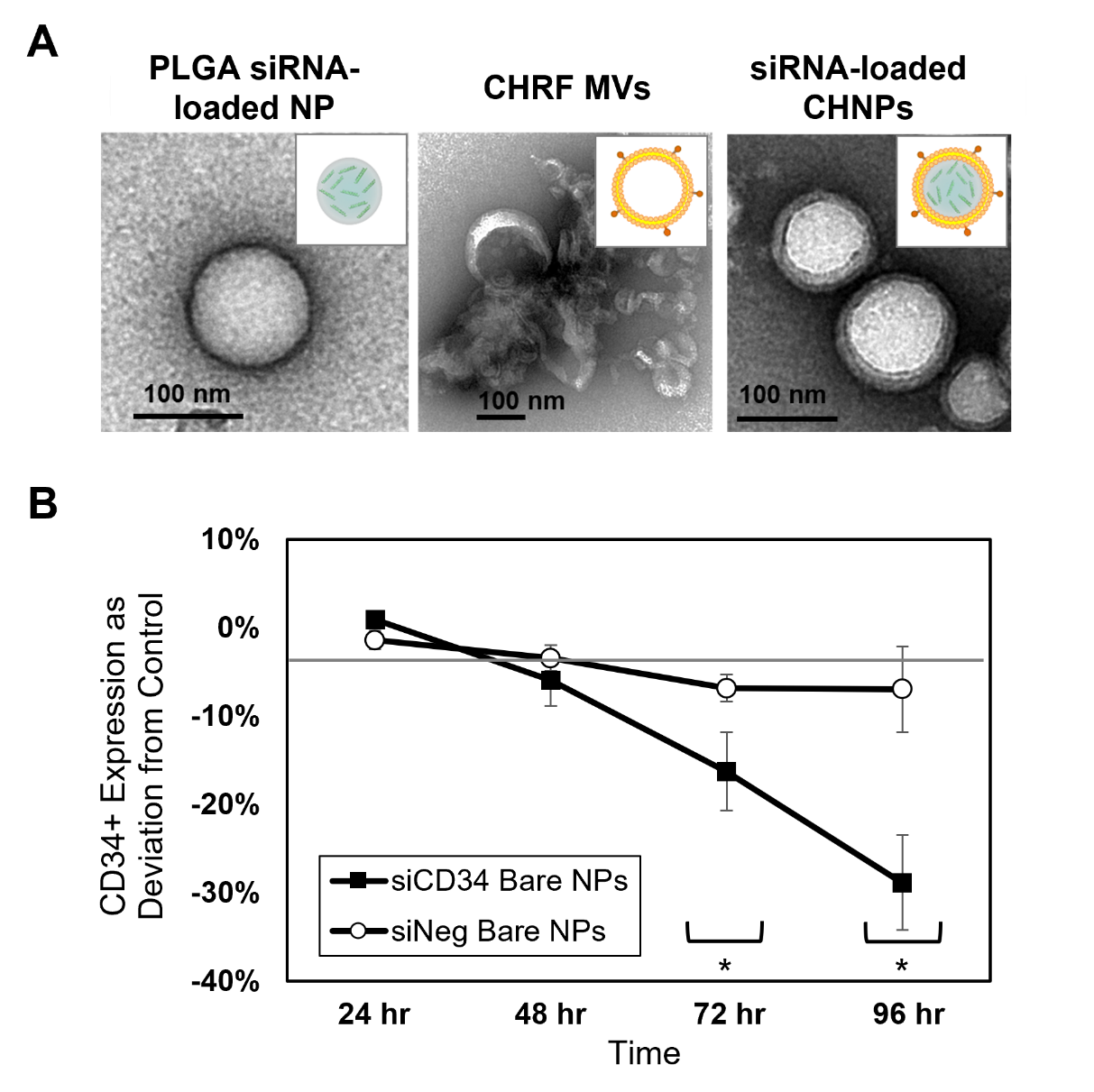


**Supplemental Figure S2. TEM microscopy of siRNA-loaded NPs and CD34 expression in HSPCs exposed to siRNA-loaded bare PLGA NPs.** (A) Transmission Electron Micrographs of PLGA siRNA-loaded NPs, CHMVs, and siRNA-loaded CHNPs. (B) CD34+ HSPCs were incubated with bare PLGA NPs loaded with siCD34 or non-targeting siRNA (siNeg) for various amounts of time, then CD34 expression was analyzed by flow cytometry. Data is shown as the deviation in CD34 expression from untreated HSPCs. Solid black squares indicate HSPCs treated with bare siCD34-loaded NPs and empty circles correspond to bare siNeg-treated HSPCs. Error bars are shown as the average of 4 replicates ± the standard error of the mean. Scale bars: 100-nm. **:p<0.05 (Student’s T-test)*

**Figure S3.**

**
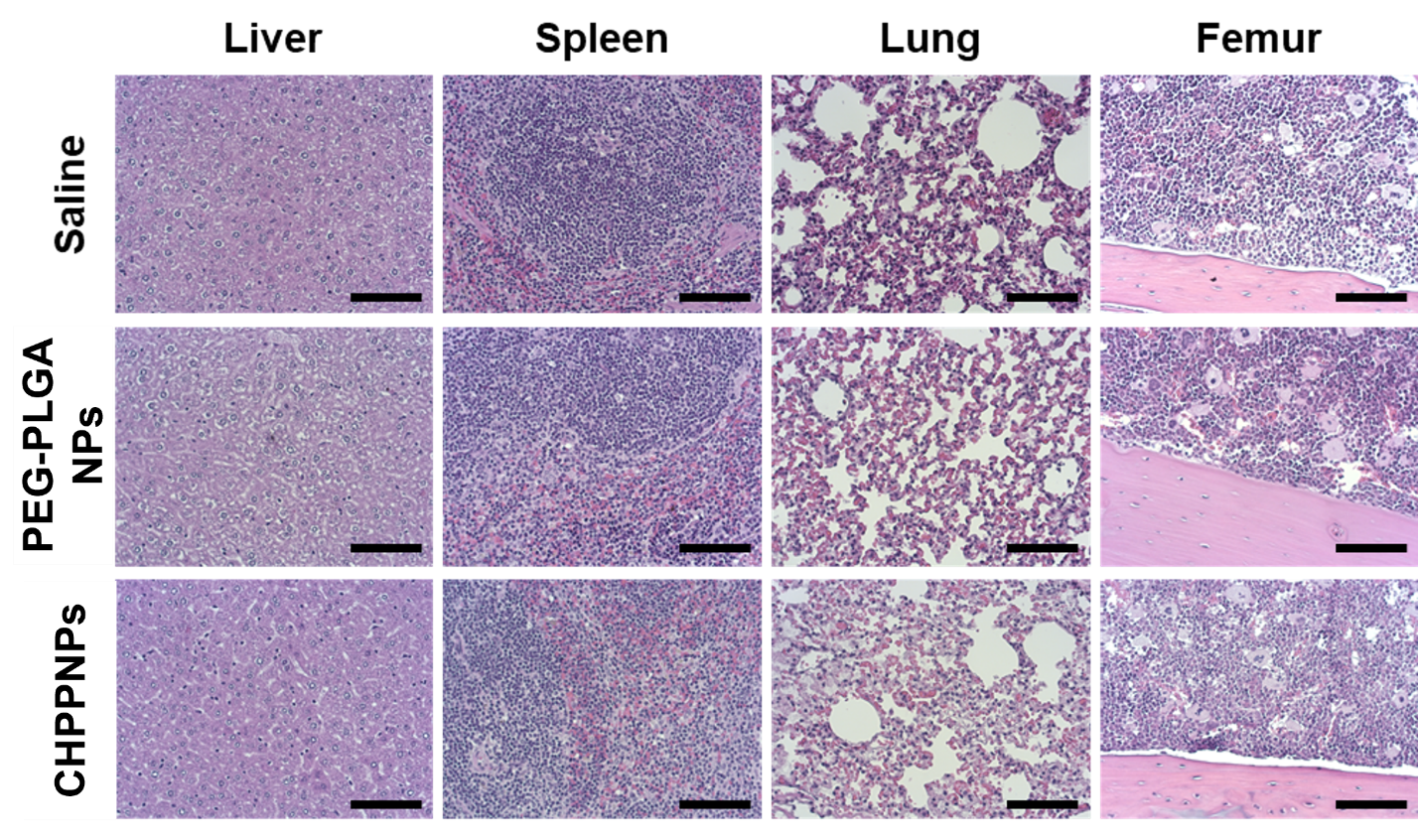
**

**Supplemental Figure S3. Representative hematoxylin and eosin (H&E) staining of tissues indicate no toxicity.** Organs from a representative mouse were paraffin embedded, sectioned, and stained with H&E to visualize any morphological changes. Images are 20X magnification. Scale bars: 100-µm.

**Figure S4.**

**
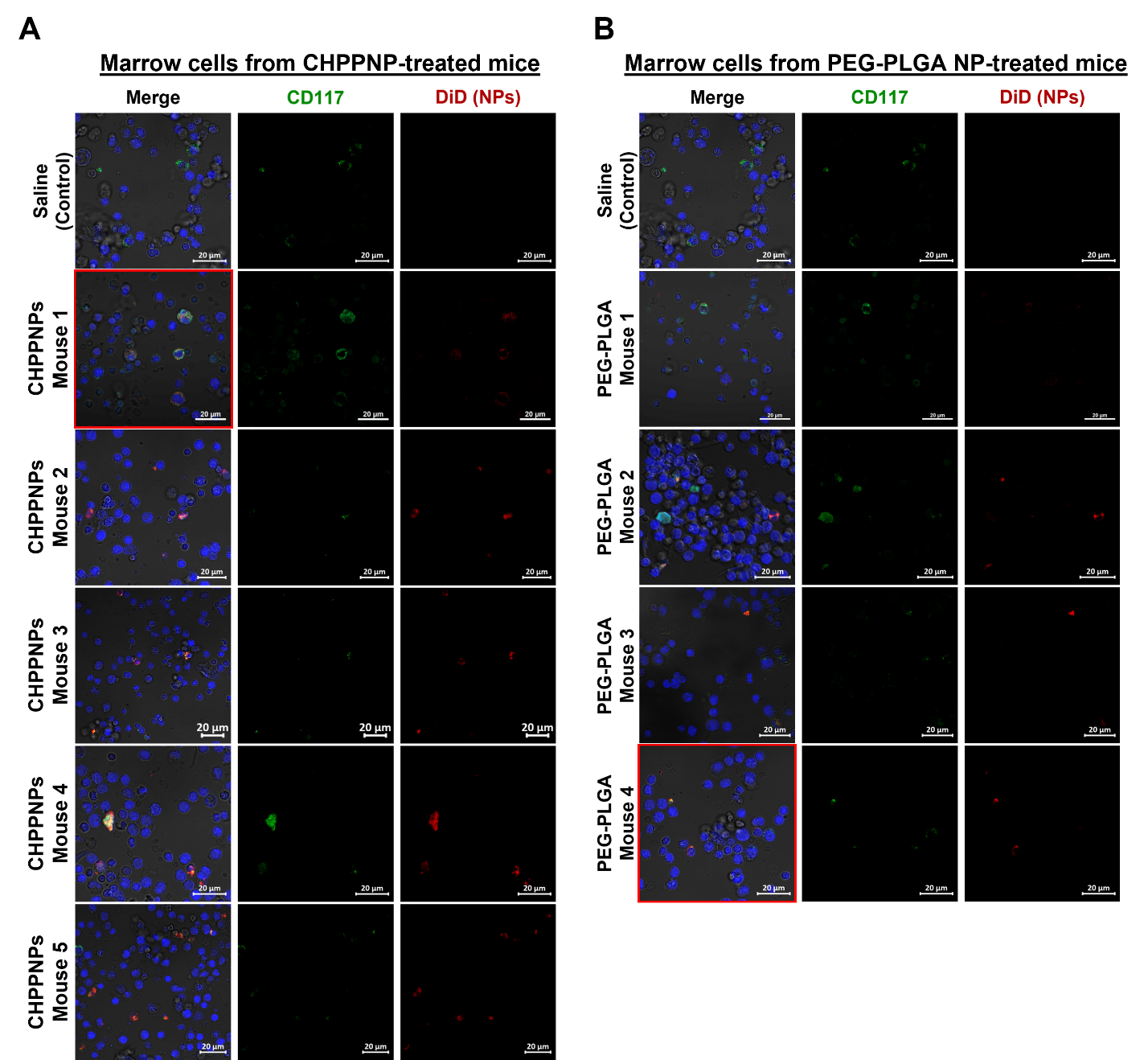
**

**Supplemental Figure S4. DiD signal presence in flushed bone marrow cells of mice treated with saline, PEG-PLGA NPs, or CHPPNPs.** Bone marrow cells were flushed from the femurs of mice treated with a saline control, (A) DiD-loaded CHPPNPs, and (B) DiD-loaded PEG-PLGA NPs. Flushed bone marrow cells were stained for CD117, a murine hematopoietic stem cell marker (green) and DAPI (blue) corresponding to the cell nuclei. The bone marrow cells were screened for presence of NPs, indicated by DiD (red). Images outlined in red were used in main text (Figure 6B). Scale bars: 20-µm.
